# Biliverdin targeting TcdB-DRBD inhibits *Clostridioides difficile* virulence and restores gut microbiota in Mongolian gerbils (Meriones unguiculatus)

**DOI:** 10.1038/s42003-025-09059-8

**Published:** 2025-11-25

**Authors:** Shuangshuang Wan, Yu Lei, Yue Jin, Runze Wang, Meng Zhang, Qikai Shi, Hui Hu, Yulei Tai, Yun Luo, Zheng Xu, Rong Kuang, Xiaojun Song, Yu Chen, Dazhi Jin

**Affiliations:** 1 School of Laboratory Medicine and Bioengineering, Hangzhou Medical College, Hangzhou, Zhejiang China; 2 Key Laboratory of Biomarkers and In Vitro Diagnosis Translation of Zhejiang province, Hangzhou, Zhejiang China; 3 Laboratory Medicine Center, Department of Clinical Laboratory, Zhejiang Provincial People’s Hospital, Hangzhou Medical College, Hangzhou, Zhejiang China; 4 School of Biotechnology and Biomolecular Sciences, University of New South Wales, Sydney, NSW Australia; 5 NMPA Key Laboratory for Animal Alternative Testing Technology of Cosmetics, Zhejiang Institute for Food and Drug Control, Hangzhou, China

**Keywords:** Infectious diseases, Bacteria

## Abstract

The incidence of *Clostridioides difficile* infection (CDI) has been rising globally in recent years. Treating CDI is complicated by antibiotic-induced disruption of the normal gut microbiota, which promotes CDI recurrence and increases the risk of therapeutic failure. We used an AI-assisted approach to screen small molecule inhibitors targeting the receptor binding domain of toxin B (TcdB). Biliverdin (BV) had strong binding affinities with all TcdB variants. In vitro results showed that BV exhibited no cytotoxic effects on cells and didn’t affect growth of *C. difficile*, yet markedly suppressed cytotoxic effects induced by TcdB1-4. Encapsulating BV in intestinal epithelial cell-derived extracellular vesicles (I-EVs) significantly recovered body weight, enhanced survival rate, reduced TcdB load, and alleviated intestinal lesions in treated gerbils. Notably, BV treatment not only restored the abundance of gut microbiota but also significantly increased the quantity of gut-beneficial *Firmicutes*. BV also exerted its anti-CDI effect by restoring the short-chain fatty acid metabolic network. Our findings indicate that BV shows promise as a natural small-molecule therapeutic that attenuates broad-spectrum TcdB-induced injuries, highlighting its potential for clinical translation in CDI treatment.

## Abstract (web summary)

Biliverdin encapsulated in intestinal epithelial cell-derived extracellular vesicles with targeted gut release inhibits *Clostridioides difficile* virulence, reduces intestinal damage, and restores gut microbiota in Mongolian gerbils, showing promise as a natural small-molecule therapeutic for CDI treatment.

## Introduction

*Clostridioides difficile*, being a Gram-positive bacterium, is associated with hospital-acquired infections and antibiotic-associated enteric diseases^1^. Prolonged and inappropriate use of broad-spectrum antibiotics, immunosuppressants, and chemotherapeutic agents can disrupt the gut microbiota^2^. This dysbiosis promotes the proliferation of *C. difficile* and increases the risk of *C. difficile* infection (CDI)^1^. Patients with CDI typically exhibit symptoms such as diarrhea and abdominal pain, along with serious complications including intestinal perforation, toxic colitis, pseudomembranous colitis, and even death^2^.

Toxin B (TcdB), a major virulence factor in CDI^3^, belongs to the large clostridial cytotoxins family and is primarily responsible for intestinal damage^4^. TcdB acts as a specific glucosyltransferase that deactivates small GTPases, including Rho, Rac and Cdc42, leading to cytoskeletal disintegration^5^ and resulting in necrosis, detachment, increased permeability, and loss of intestinal barrier function in intestinal mucosal cells^6^. TcdB contains four functional domains: an N-terminal glucosyltransferase domain (GTD), a cysteine protease domain (CPD), a central delivery and receptor binding domain (DRBD), and a C-terminal combinatorial repeat oligopeptide (CROP) domain^7^. Through its DRBD and CROP domains, TcdB binds to host cell surface receptors and enters host’s intestinal cells via endocytosis^8^. Endosomal acidification induces conformational changes in TcdB, promoting pore formation in the DRBD and translocation of GTD and CPD into the cytosol^9^. TcdB variants exhibit receptor specificity: variants 1 and 3 bind FZDs 1, 2, and 7 as well as CSPG4, while variants 2 and 4 interact with TFPI^10,11^. The DRBD of TcdB is critical not only for receptor binding but also for mediating the intracellular delivery of the toxin, making it essential to TcdB’s pathogenicity.

Currently, antibiotics such as metronidazole, vancomycin, and fidaxomicin remain the primary treatment for CDI^12^. Nonetheless, up to 25% of patients with CDI treated with metronidazole and vancomycin experienced a recurrence of the infection within 30 days of treatment. Of them, ~45–65% experienced a further recurrence^13^. Fidaxomicin was shown to be superior than vancomycin in reducing CDI recurrence and had a minimal impact on the gut microbiota^13^. Nevertheless, its higher cost raises concerns about economic viability, potentially restricting its application in the management of CDI^14^. Antibiotic use is associated with CDI recurrence, adverse effects, and disruption of the gut microbiota^15^. Over the past decade, small molecule inhibitors and neutralizing antibodies have emerged as promising non-antibiotic therapeutic options for CDI^16^. However, there remains an urgent need for novel strategies-particularly non-antibiotic agents that directly target virulence factors-to reduce antibiotic dependence and minimize host harm^17^.

Our study used an artificial-intelligence-assisted approach to screen natural small- molecule candidates targeting the structural DRBDs of all TcdB variants. The interaction between the candidates and TcdB-DRBD was investigated through molecular docking. The optimal candidates were validated in vitro and in vivo to prevent TcdB variants-induced cell rounding and to alleviate the symptoms of CDI. Furthermore, the small molecule’s effects on the gut microbiota were also analyzed using a CDI animal model established in Mongolian gerbils, which possess several human-like features and demonstrated gut microbiota alteration patterns similar to humans following *C. difficile* challenge^18^. Our study identified a promising non-antibiotic and natural small-molecule therapeutic agent for treating CDI by alleviating pathological injuries induced by broad-spectrum TcdB.

## Results

### Molecular docking and molecular dynamics simulation

**Fig. 1:**
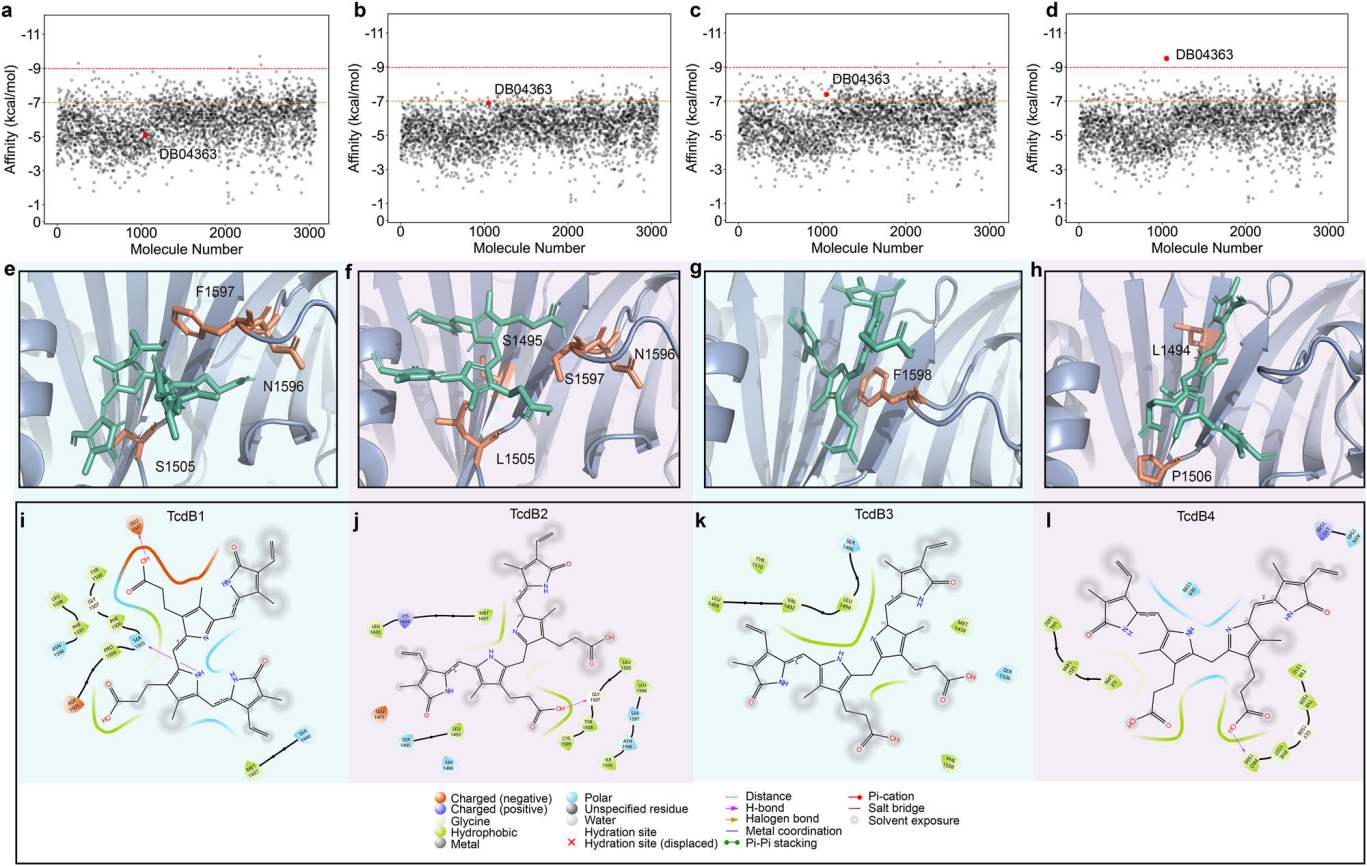
Results of screening and molecular docking. Scatter plots with binding energies of small molecule compounds screened against TcdB1-4, red dot refer to BV while gray dots refer other compounds in DrugBank (**a**–**d**). BV and TcdB1-4 complex structures for AutoDock Vina simulations (**e**–**h**). BV is shown in green, and key amino acid residues are in yellow. The non-covalent interaction between BV and TcdB1-4 (**i**–**l**) residues at the molecular level.

Binding affinity values better than −9.0, −7.0, and −5.0 kcal/mol indicated strong, moderate, and weak binding activities, respectively, between the ligand and the target^19^. Molecular docking analysis revealed distinct interaction patterns between four TcdB subtypes (TcdB1-4) and the target compound DB04363, with significant variations observed in binding free energy, critical binding sites, and in termolecular force distributions. Molecular docking demonstrated a clear binding affinity with TcdB4 (−9.6 kcal/mol), TcdB3 (−7.4 kcal/mol), TcdB2 (−6.9 kcal/mol), and TcdB1 (−5.1 kcal/mol), indicating the strongest binding interaction between DB04363 and TcdB4 (Fig. 1a–d and Supplementary Data 1). Structural visualization using PyMOL elucidated the binding conformations of DB04363 with TcdB1-4 (Fig. 1e–h). Subsequent analysis with Schrödinger’s 2D Sketcher module demonstrated that H-bond, electrostatic complementarity and hydrophobic interactions predominantly mediated the binding of DB04363 and TcdB1-4 (Fig. 1i–l). To evaluate the broad-spectrum potential of DB04363, TcdB5-8 variants were also analyzed. The results revealed differential binding affinities (Table 1), binding sites, and intermolecular force distributions across all tested TcdB variants (Supplementary Fig. 1). DB04363 in DrugBank is Mesobiliverdin IV alpha. Mesobiliverdin (MBV) and biliverdin IXα (BV) have nearly identical structures except for the reduction of BV’s two vinyl groups to ethyl groups in MBV (eliminating two carbon-carbon bonds), hereafter, both compounds are collectively referred to as biliverdin (BV).

**Table 1:** Results of docking of DB04363 with other TcdB variants

Variant	Affinity (kcal/mol)	Binding site
TcdB1	−5.1	F1597, N1596, S1505
TcdB2	−6.9	L1494, P1506
TcdB3	−7.4	F1598
TcdB5	−7.1	G1508, F1507, S1506
TcdB6	−6.9	M1437, S1440, D1501
TcdB7	−6.7	E1432, M1437
TcdB8	−7.1	S1495, H1596

Possessing the highest binding affinity, the TcdB4-BV complex, was investigated through 100 ns comprehensive molecular dynamics (MD) simulations to assess its temporal stability and structural adaptation. The root-mean-square deviation (RMSD) indicated that TcdB4 reached a stable conformation(Supplementary Fig. 2a). Root-mean-square fluctuation (RMSF) of TcdB4 analysis confirmed minor conformation modification of TcdB4 where binding to DB04363 (Supplementary Fig. 2b). Binding free energy decomposition analysis using the MM/PBSA^20^ method yielded a Gibbs free energy of approximately −40 kcal/mol (Supplementary Fig. 2c), indicating a strong interaction.

### UV absorption and SPR analysis of the TcdB2-BV interaction

**Fig. 2:**
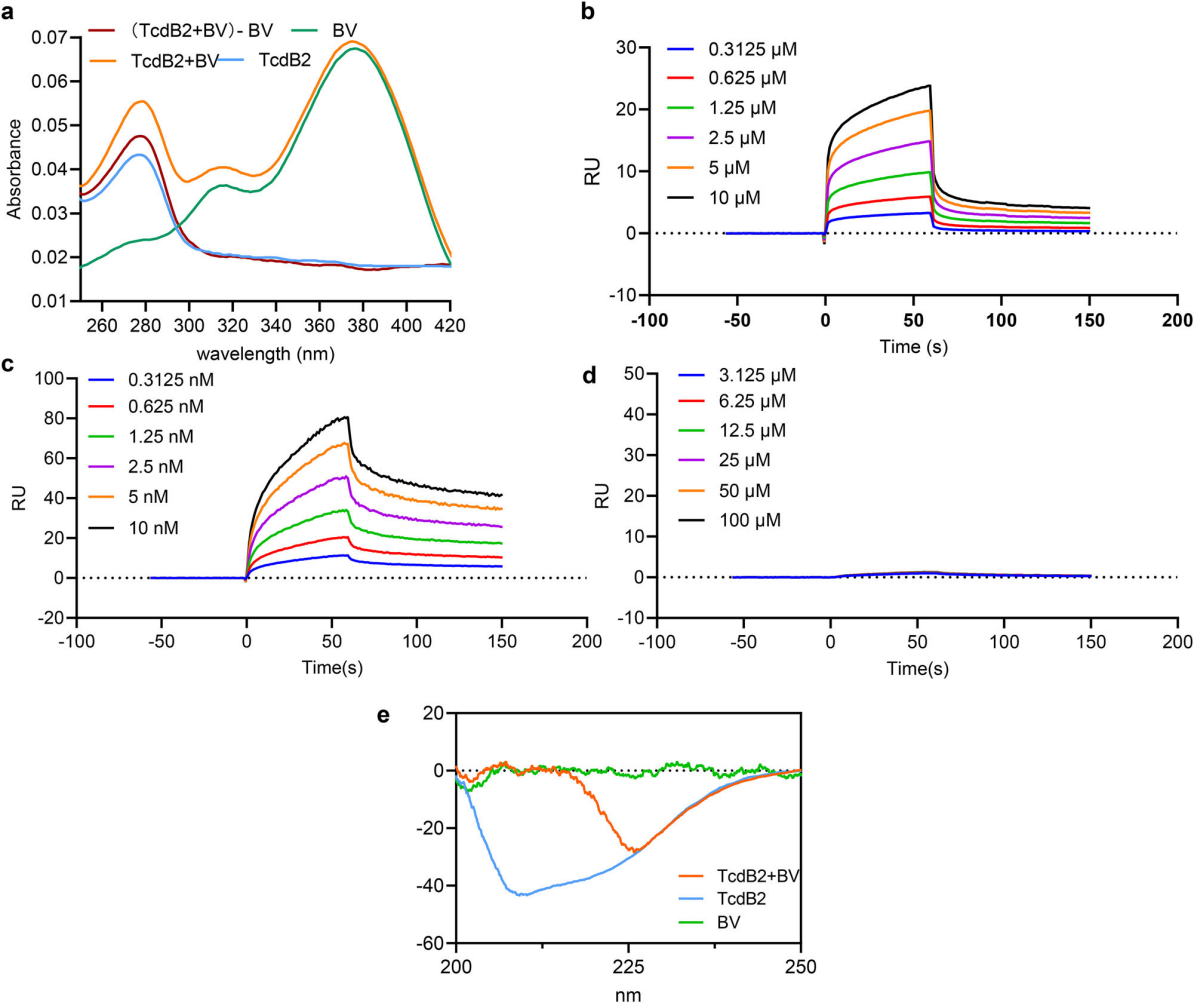
Validation of the interaction between BV and TcdB2. **a** UV absorption spectra of BV after incubation with TcdB2. **b** The interaction between BV and TcdB2 was determined by SPR. **c**, **d** SPR binding curves of E3 and vancomycin at different concentration gradients with TcdB2, respectively. **e** CD Spectra of TcdB, BV, and TcdB + BV.

It has been demonstrated that TcdB2, a key virulence factor produced by hypervirulent *C. difficile* ribotype 027 (RT027) strains^21^, often leads to severe clinical outcomes with high recurrence rates, high morbidity and mortality^22^. Therefore, TcdB2, as an example, was used to analyze the interaction between BV and TcdB variants. The specific absorption peaks of the TcdB2 and BV interaction in the UV spectra were identified at 280 nm and 375.5 nm, respectively. When BV was combined with TcdB2, there was a marked increase in the absorption peak at 280 nm (Fig. 2a). The spectra of TcdB2 alone were different from that of [(TcdB2 + BV) - BV]. The results of SPR showed a concentration-dependent binding of TcdB2 to BV (Fig. 2b). The strongest binding between BV and TcdB2 (50 μg/mL) was observed at a BV concentration of 10 μM, and the dissociation constant KD was 2.53 × 10^−6 ^M. The TcdB-neutralization antibody E3 prepared in our laboratory as a positive control bound firmly to TcdB2, and vancomycin as a negative control showed no detectable binding to TcdB2, confirming the specificity of the interaction between BV and TcdB2 (Fig. 2c, d). The circular dichroism (CD) analysis also revealed a significant specific interaction between BV and TcdB2 (Fig. 2e). At 222 nm, the signal intensity (−17.47 mdeg) of TcdB2 combined with BV was much higher than the theoretical sum value (BV: 1.015 + TcdB2: −34.66 = −33.64 mdeg), indicating that BV induced the unwinding of the α-helical structure of TcdB2, demonstrated that TcdB2 conformational changes occurred when BV was specifically bound.

### BV protecting TcdB1-4 induced cell rounding but not affecting the growth of *C. difficile*

**Fig. 3:**
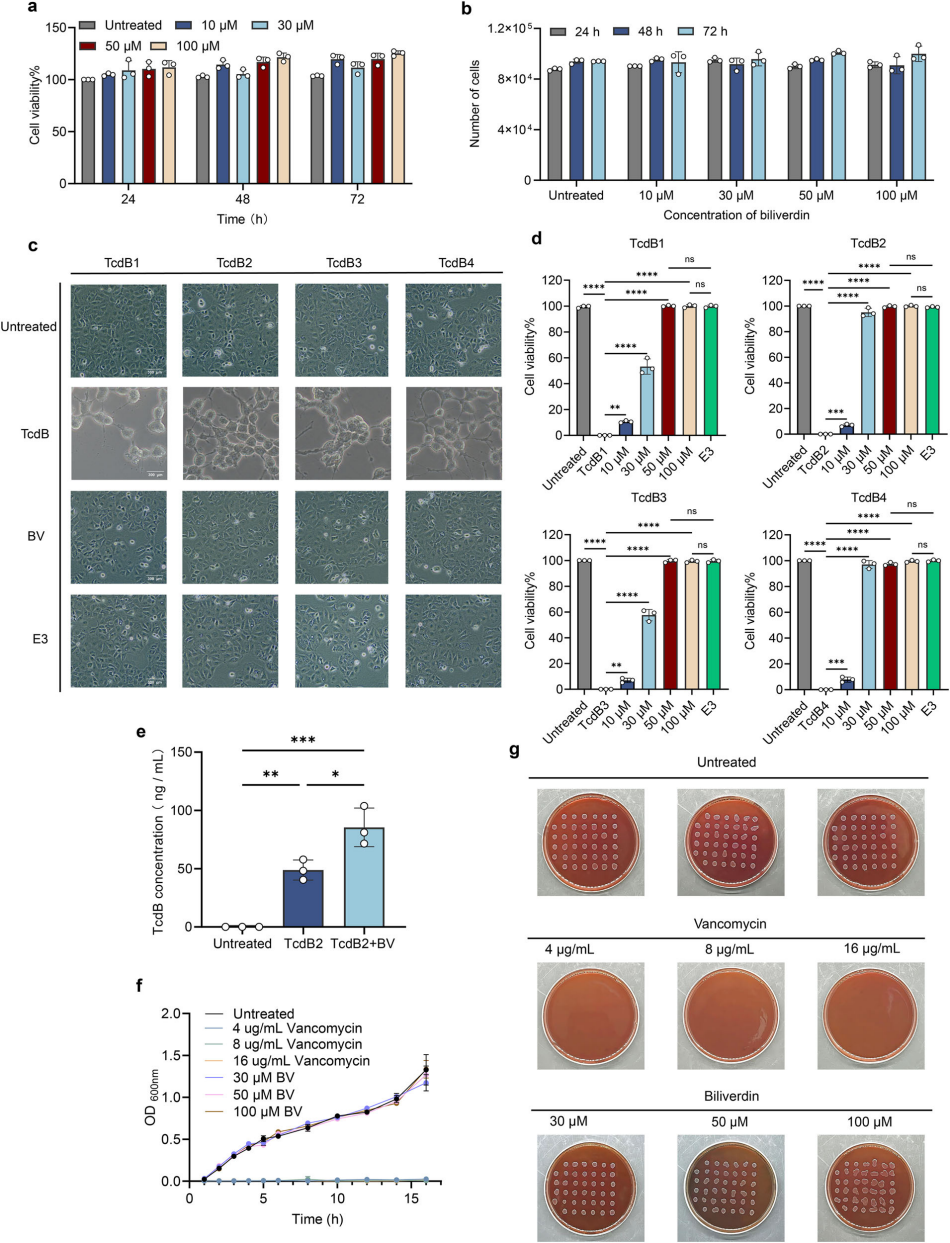
In vitro results of BV. **a**, **b** Stimulation of Caco-2 cells with 10 μM, 30 μM, 50 μM and 100 μM BV at 24 h, 48 h and 72 h (*n* = 3). **c** TcdB1-4 treated CaCo-2 cells with and without BV, E3 was a TcdB neutralization antibody used as a positive control (*n* = 3) (scale bar = 300 μm). **d** The protective effects of BV at different concentrations were quantitatively measured by the CCK-8 assay (*n* = 3). **e** Measurement of TcdB levels in cell culture supernatants (*n* = 3). **f** Growth curves of the *C. difficile* strain (ATCC BAA-1870, ST1/RT027) treated by different concentrations of vancomycin and BV (*n* = 3), respectively. **g** Comparative analysis on the growth of the *C. difficile* (ATCC BAA-1870, ST1/RT027) strain on agar plates with different concentrations of vancomycin and BV at 48 h (*n* = 3), respectively. * *p* < 0.05, ** *p* < 0.01, *** *p* < 0.001, **** *p* < 0.0001, ns *p* > 0.05.

The results showed that there was no difference in the growth of CaCo-2 cells compared to the untreated cells when BV was administered at four different concentrations at different time points, indicating that BV had no cytotoxicity and did not inhibit cell growth (Fig. 3a, b). To explore whether BV protects CaCo-2 cells against TcdB-induced cytopathic effects, cells were exposed to TcdB1-4 (1 pM) with and without BV, and the TcdB-neutralization antibody E3 was used as a positive control for evaluation of BV. Morphological analysis showed that all TcdB1-4 variants induced rounding and shrinkage of CaCo-2 cells within 24 h. However, no significant alterations in cell morphology were observed when CaCo-2 cells were treated for 24 h with TcdB1-4 incubating with BV. The results were consistent with those of the positive control, indicating that BV inhibited TcdB-induced cell rounding, which was comparable to the expected outcome (Fig. 3c). The CCK-8 results also showed that each of TcdB1-4 alone reduced viability to 10–20% of CaCo-2 cells in comparison to the untreated cells, while 10–100 µM of BV co-treatment restored viability to 50–100% of cells. No significant differences were found in cell viability induced by TcdB1-4 with BV and E3, respectively (*p* > 0.05) (Fig. 3d). In addition, the results showed that TcdB concentration was significantly higher in the cell culture supernatant after BV treatment in comparison to no BV treatment (*p* = 0.0141) (Fig. 3e), indicating that BV interacted with TcdB and impeded its entry into cells.

In addition, the growth curves showed that BV, unlike vancomycin, did not significantly inhibit the growth of the *C. difficile* strain (ATCC BAA-1870, sequence type [ST]1/RT027). A similar result was also observed when *C. difficile* was cultured on agar plates with different concentrations of BV (Fig. 3f, g).

### Characterizations and in vitro drug release of I-EV-BV

**Fig. 4:**
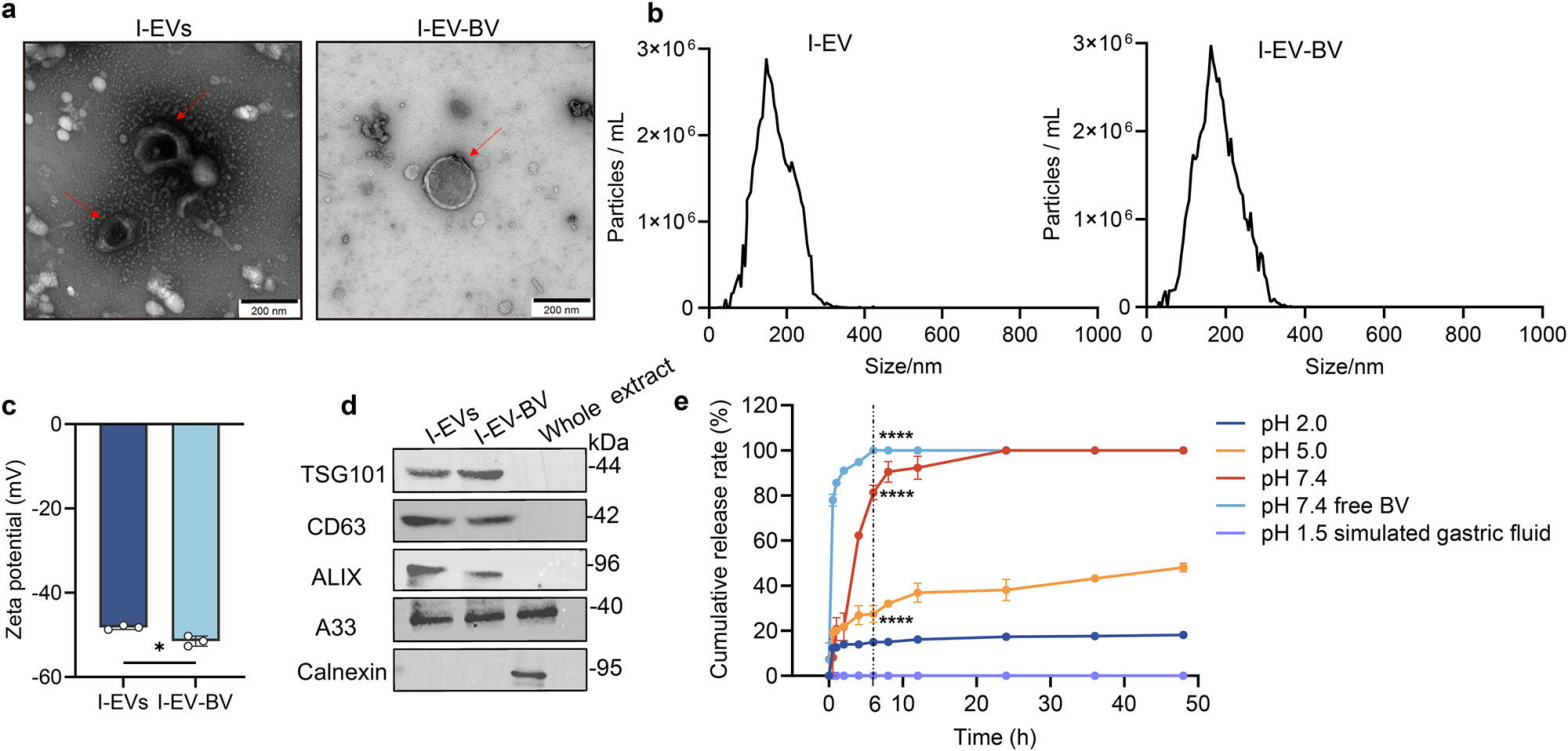
Characterization of I-EVs and I-EV-BV. **a** Images of I-EVs and I-EV-BV were visualized by TEM (scale bar = 200 nm). **b** Size distribution of I-EVs and I-EV-BV measured by ZetaVIEW. **c** Zeta potential analysis of I-EVs and I-EV-BV. **d** I-EVs and I-EV-BV were positively identified by Western blotting. **e** BV release from I-EV-BV and free BV at different pH values and in gastric juice (with pepsin). The pH 7.4 free BV: BV not loaded into I-EVs (*n* = 3). * *p* < 0.05, **** *p* < 0.0001.

Cup-shaped and concave-shaped extracellular vesicles were isolated from intestinal epithelial cells (I-EVs) and observed via transmission electron microscopy, however I-EVs became relatively round after loading with BV (I-EV-BV) (Fig. 4a). The particle size distribution of I-EVs ranged from 100 to 200 nm (mean: 147.5 nm), and however, the I-EV-BV showed a larger particle size than that of I-EVs, with a size distribution of 100–300 nm (mean: 162.5 nm) (Fig. 4b). In addition, zeta potential analysis showed that the surface charge of I-EV-BV was significantly lower than that of unloaded I-EVs (*p* = 0.0125), indicating that BV loading increased the stability of the I-EVs (Fig. 4c). The EV markers, CD63, TSG101, and ALIX were positively identified in the extracted I-EVs and prepared I-EV-BV, both of which also contained the intestinal epithelial cell-specific protein A33 but not calnexin, demonstrating that I-EVs and I-EV-BV originated from intestine epithelial cells with similar protein composition (Fig. 4d and Supplementary Fig. 3). Moreover, the result on the calculated DL of I-EV-BV showed that ~43.4% of BV was loaded into the I-EVs. Subsequently, the in vitro release profiles of I-EV-BV were evaluated at pH values of 7.4, 5.0 and 2.0, corresponding to physiological conditions. Our results indicated that at 6 h, the release rate of I-EV-BV at pH 7.4 was 81.2%, and at pH 5.0 it was 27.4%, which was significantly higher compared to the release rate at pH 2.0 (15%) (*p* < 0.0001) (Fig. 4e). At the same time, the release rate of free BV has reached 100% (Fig. 4e) (*p* < 0.0001). The peak release of I-EV-BV occurred around 24 h. The above results indicate that I-EV-BV exhibited the properties of a sustained-release drug delivery system. Furthermore, the I-EV-BV drug delivery system demonstrated minimal loss in the stomach, with no drug release observed when placed in artificial gastric fluid containing pepsin (pH 1.5), indicating nearly complete targeting of the intestinal tract.

### I-EV-BV exhibiting a protective effect on the CDI animal model

**Fig. 5:**
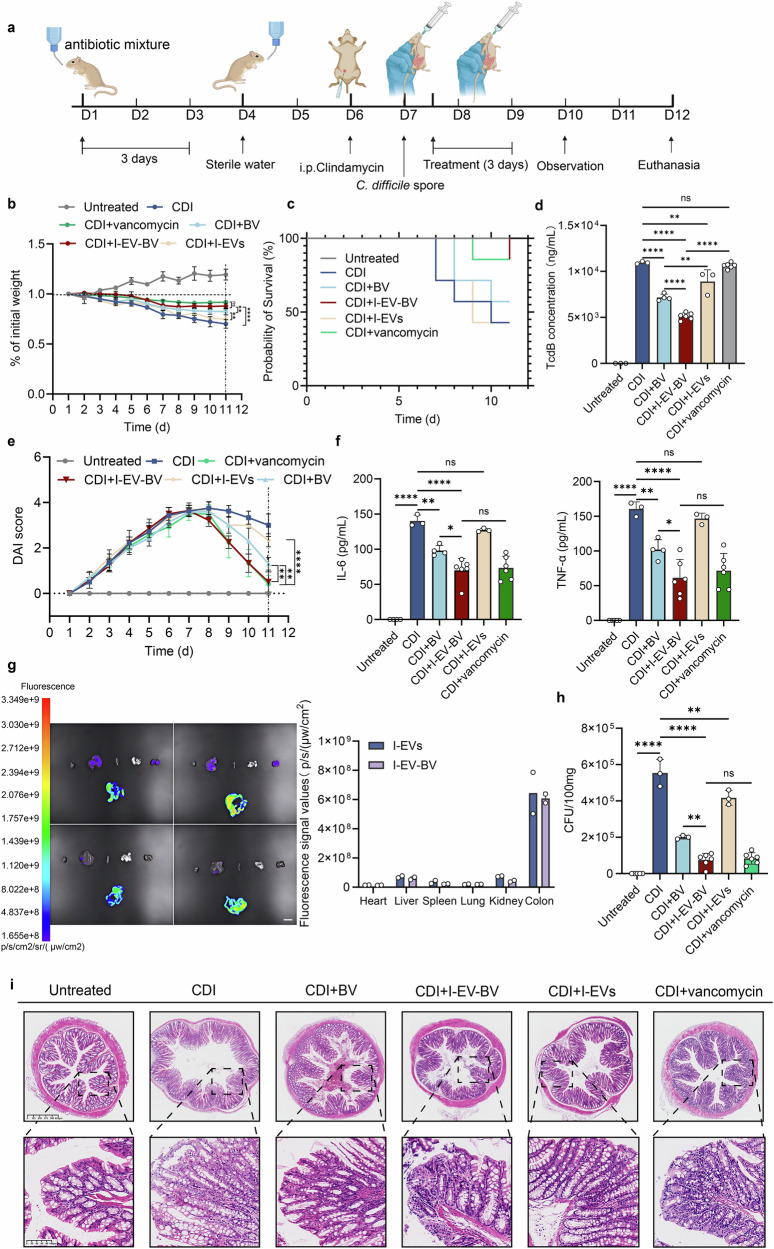
In vivo results of BV. **a** Sequence of CDI animal model construction. (Untreated, *n* = 5; CDI, *n* = 7; CDI + BV, *n* = 7; CDI + I-EV-BV, *n* = 7; CDI + I-EVs, *n* = 7; CDI+ vancomycin, *n* = 7). **b** After being challenged by *C. difficile* spores, the Mongolian gerbils were weighed, and the change in body weight was recorded at regular intervals every day, and the ratio was calculated according to the body weight of each gerbil on day 1. **c** Survival of Mongolian gerbils before and after challenge by *C. difficile* spores. **d** TcdB concentration measured by ELISA in animal intestinal contents (*n* = 3–6 per group). **e** The DAI scores of different groups (*n* = 5-7 per group). **f** Comparison of proinflammatory cytokines IL-6 and TNF-α in colonic tissues among different groups (*n* = 3–6 per group). **g** Fluorescence imaging of organs and quantitative analysis of fluorescence signals in different organs in the I-EVs (upper) and I-EV-BV (lower) groups (*n* = 2). Scale bar = 1 cm. **h** The *C. difficile* load in the intestinal contents of different groups (*n* = 3–6 per group). **i** HE staining of the intestines of the five groups of Mongolian gerbils. Scale bar: 625 µm. The scale bar for the zoomed-in images: 100 µm. * *p* < 0.05, ** *p* < 0.01, **** *p* < 0.0001, ns *p* > 0.05.

The CDI model of Mongolian gerbils was constructed as described in Fig. 5a^18^. Following *C. difficile* spore (ATCC BAA-1870) challenge, Mongolian gerbils exhibited symptoms of wet tails, lethargy, and significant diarrhea, with a notable decrease in body weight. However, the I-EV-BV treated group experienced a recovery in body weight on the 9th day compared to the CDI group, indicating the targeted therapeutic efficacy of I-EV-BV in the gut (Fig. 5b) (*p* < 0.0001). Survival analysis revealed that 85.7% of gerbils in the I-EV-BV treated group still survived on Day 11 post-*C. difficile* spores-induced gerbil model (Fig. 5c). The TcdB concentration in the intestinal contents of the I-EV-BV treated group was ~5.1 μg/μL, which was significantly lower than that in the CDI group (10.95 μg/μL) (*p* < 0.0001) (Fig. 5d). The Disease Activity Index (DAI) scores indicated a steady decline in symptoms for both the BV and I-EV-BV treated groups from Day 8, with a notable absence of diarrhea in the I-EV-BV treated group on Day 11 (Fig. 5e). The expression levels of pro-inflammatory cytokines IL-6 and TNF-α revealed a marked elevation in the colon tissue in the CDI group, whereas the I-EV-BV treatment group exhibited a significant reduction in their levels (*p* < 0.0001) (Fig. 5f). Furthermore, there were no significant differences found on body weight, DAI, and expression levels of pro-inflammatory cytokines between vancomycin and I-EV-BV treated groups. In vivo biodistribution analysis of both DiI-labeled I-EVs and I-EV-BV showed distinct organ-specific accumulation. Fluorescent imaging of gerbil organs (heart, liver, spleen, lungs, kidneys, and intestines) presented robust fluorescence signals in intestinal tissues, moderate accumulation in the liver and spleen, and minimal detectable fluorescence in other organs (Fig. 5g), collectively indicating preferential targeting of intestinal tissues by the I-EVs. Quantitative intestinal contents analysis showed high *C. difficile* colonization in the CDI group (Fig. 5h). Furthermore, I-EV-BV treatment significantly reduced *C. difficile* load in comparison with CDI (*p* < 0.0001). Histological evaluation of the colon by H&E staining also showed that the CDI group exhibited epithelial damage, irregular arrangement, and a significant reduction in the number of cup cells in the crypts with neutrophil infiltration. On the contrary, only irregular arrangements of myofibroblasts and loose cellular arrangements were observed in the I-EV-BV treated group (Fig. 5i).

### Impact of BV treatment on gut microbiota

**Fig. 6:**
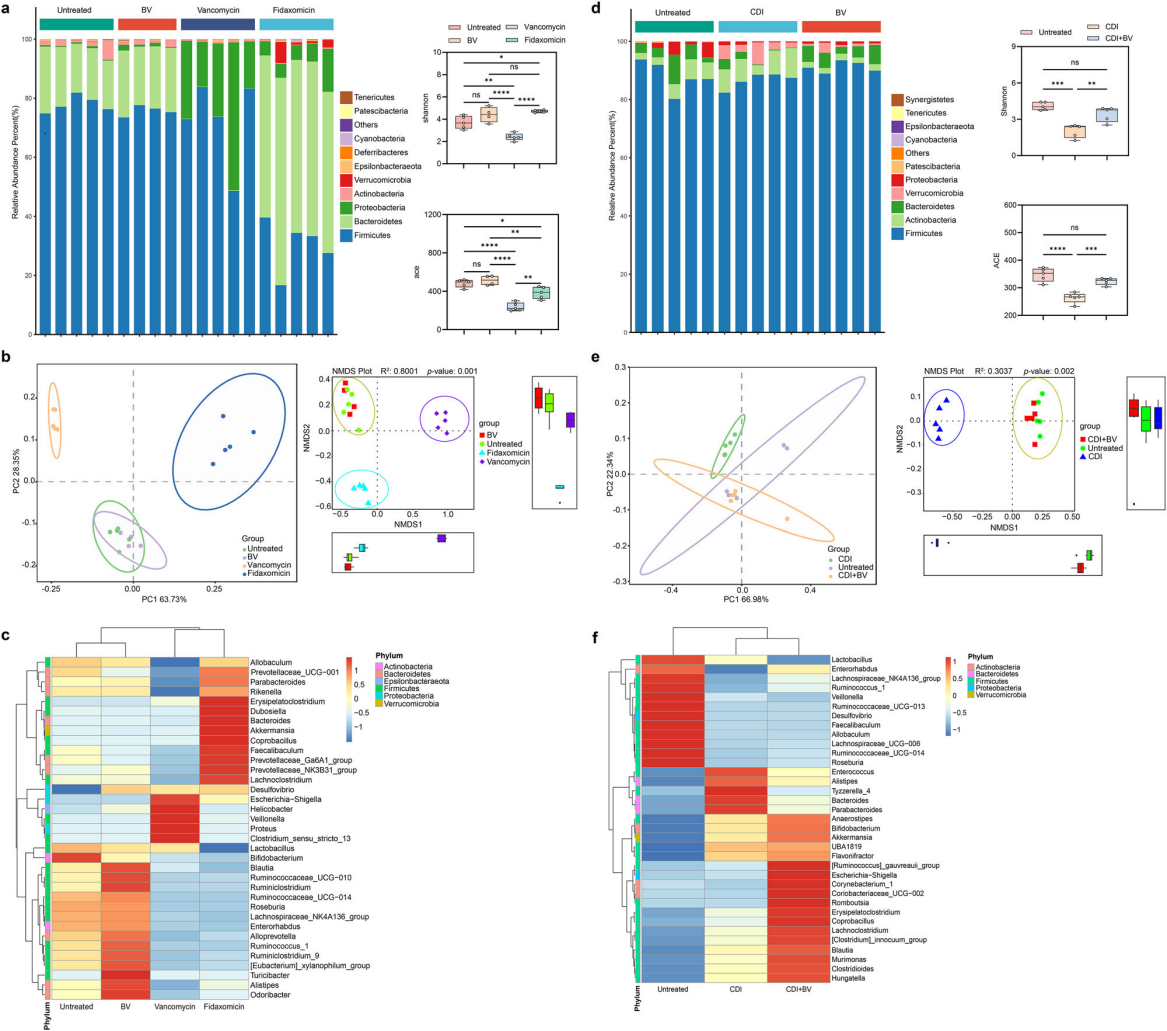
Analysis of the gut microbiota by 16S rDNA sequencing. The gut microbiota of gerbils was compared by 16S rDNA deep sequencing analysis after 3 days of treatment in the absence of CDI **a**–**c** (*n* = 4–5 per group) and in the presence of CDI **d**–**f** (*n* = 5), respectively. The top 10 species with the maximum abundance are ranked at each taxonomic level (Phylum, Class, Order, Family, Genus). Numbers indicate data for individual gerbil in each group; Shannon and ACE Index are used to analyze differences in species diversity and abundance between groups. PCoA analysis was performed, and the combination of principal coordinates with the largest contribution was selected for presentation. The results of the NMDS (Non-Metric Multi-Dimensional Scaling) analysis based on the level of OTUs are shown, and the figure of each point represents one sample. A heat map of the relative abundance of the top 35 genera in the gerbil gut microbiota. * *p* < 0.05, ** *p* < 0.01, *** *p* < 0.001, **** *p* < 0.0001, ns *p* > 0.05.

The composition and diversity of the Mongolian gerbil gut microbiota were evaluated after 3 days of BV treatment in comparison to vancomycin and fidaxomicin treatment. As shown in Fig. 6a, vancomycin significantly altered the structure of the gut microbiota, with a decrease in the abundance of *Bacteroides* and an increase in the relative abundance of *Proteobacteria* following 3 days of oral administration. Furthermore, the Shannon and ACE index in the BV treated group compared to the untreated group were not significantly different (*p* > 0.05) (Fig. 6a). Meanwhile, the effect of BV on the diversity and abundance of gut microbiota in Mongolian gerbils was less than that of vancomycin (*p* < 0.0001). Notably, the Shannon index in the BV treated group was not significantly different compared to the fidaxomicin treated group (*p* = 0.7588) (Fig. 6a). However, from the ACE index, the abundance of the BV treated group was higher than that of the fidaxomicin treated group (*p* = 0.0075) (Fig. 6a). The Principal Co-ordinates analysis (PcoA) and NMDS analysis also demonstrated that the BV treated group were largely concordant with the untreated microbiota, indicating that the differences between both microbial communities were minimal (Fig. 6b). It was observed that the levels of *Blautia*, *Ruminococcaceae*, *Ruminiclostridium*, *Alloprevotella*, *Ruminococcus*, *Eubacterium*, *Turicibacter*, *Roseburia*, *Alistipes*, *Odoribacter* and other genera of bacteria increased in the BV treated group compared to those from the untreated group, all of which belonged to the genus of *Firmicutes* (Fig. 6c). Therefore, the BV treatment did not change the overall diversity or the abundance of the gut microbiota significantly, implying that it maintained the balance of the gut microbials.

CDI induced a marked alteration in the gut microbiota profile, with a notable decrease in *Bacteroides* and a corresponding increase in *Verrucomicrobia* (Fig. 6d). Subsequent analysis using the Shannon index and ACE index showed no significant difference in microbial community diversity between the BV treated group and the untreated group (*p* > 0.05). PCoA and NMDS analyses also showed that the BV treated group and the untreated group had similar microbial communities (Fig. 6e). Compared to the untreated group, *Lactobacillus*, *Enterorhabdus*, *Lachnospiraceae*, *Ruminococcus*, *Veillonella*, *Ruminococcaceae*, *Desulfovibrio*, *Faecalibaculum*, *Allobaculum*, *Lachnospiraceae*, *Roseburia* and other genera of bacteria were significantly reduced in the CDI group (Fig. 6f). After BV treatment, it was clear that *Ruminococcus, Escherichia, Corynebacterium, Coriobacteriaceae, Romboutsia, Erysipelatoclostridium, Coprobacillus, Lachnoclostridium, Blautia, Murimonas, Hungatella* and other genera of bacteria were increased in comparison to the CDI group (Fig. 6f). However, it is important to note that BV treatment also increased the genus of gut-beneficial *Firmicutes*, which was consistent with the previous result.

### KEGG pathway enrichment analysis

In the absence of CDI, KEGG analysis showed that three distinct metabolic pathways including riboflavin metabolism, fat digestion and absorption, which mean values were significantly higher in the BV treated group compared to the untreated group (Supplementary Fig. 4a). Whereas, after *C. difficile* challenge, the CDI and BV treated groups indicated statistical significance in 52 metabolic pathways (Supplementary Fig. 4b). The differences on metabolic pathways between these two groups mainly focused on citrate cycle, oxidative phosphorylation, pyruvate metabolism, fatty acid degradation, glycine, serine and threonine metabolism. In the CDI group, the mean values of the citrate cycle, oxidative phosphorylation, and pyruvate metabolism were significantly higher compared to those in the BV treated group. Conversely, the mean values of cysteine and methionine metabolism, valine, leucine and isoleucine biosynthesis and the insulin signaling pathway were notably lower than those in the BV treated group.

### Effect of BV treatment on short-chain fatty acid levels

**Fig. 7:**
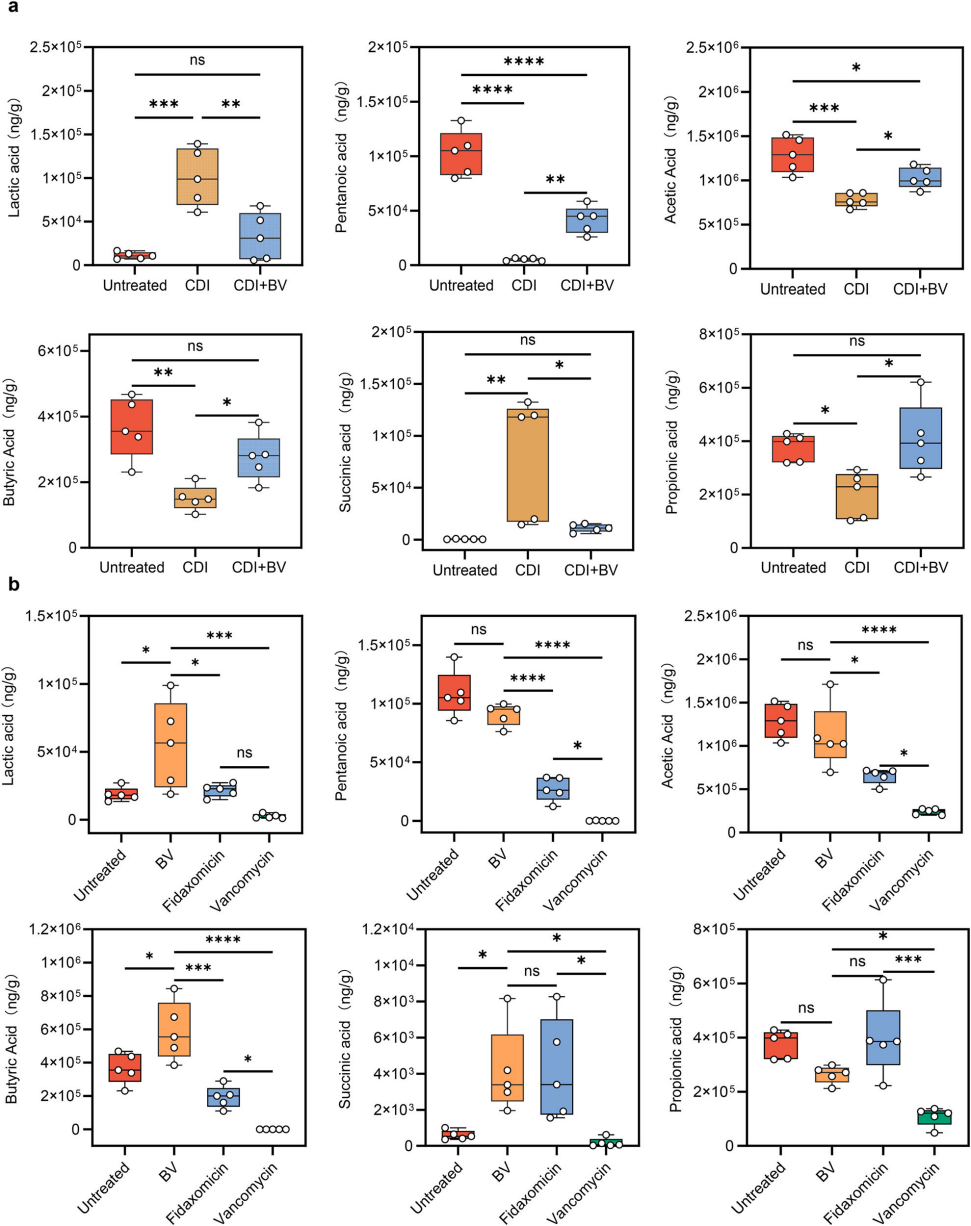
Quantitative detection of SCFAs in intestinal contents. Results of SCFAs in different groups infected with *C. difficile* **a** (*n* = 5) and without *C. difficile* **b** (*n* = 5). * *p* < 0.05, ** *p* < 0.01, *** *p* < 0.001, **** *p* < 0.0001, ns = *p* > 0.05.

The short-chain fatty acids (SCFAs) profile in the gut was examined before and after BV treatment in the gerbil CDI model (Fig. 7a). After *C. difficile* challenge, a characteristic SCFAs disruption pattern was observed, with significant decrease in butyric acid, pentanoic acid, propionic acid, and acetic acid, and significant increase in succinic acid and lactic acid. After 3 days of BV treatment, the trends were opposite to those in the CDI group. Butyric acid, pentanoic acid, propionic acid, and acetic acid significantly increased while succinic acid and lactic acid significantly decreased (Fig. 7a). These findings highlighted BV’s capacity to restore SCFAs homeostasis and stabilize intestinal microecology. In the non-CDI animal models, BV treatment significantly increased butyric acid levels. However, the levels of pentanoic acid, propionic acid, and acetic acid remained unchanged versus the untreated group (Fig. 7b).

### ADMET assessment pharmacokinetic profile analysis of BV

**Fig. 8:**
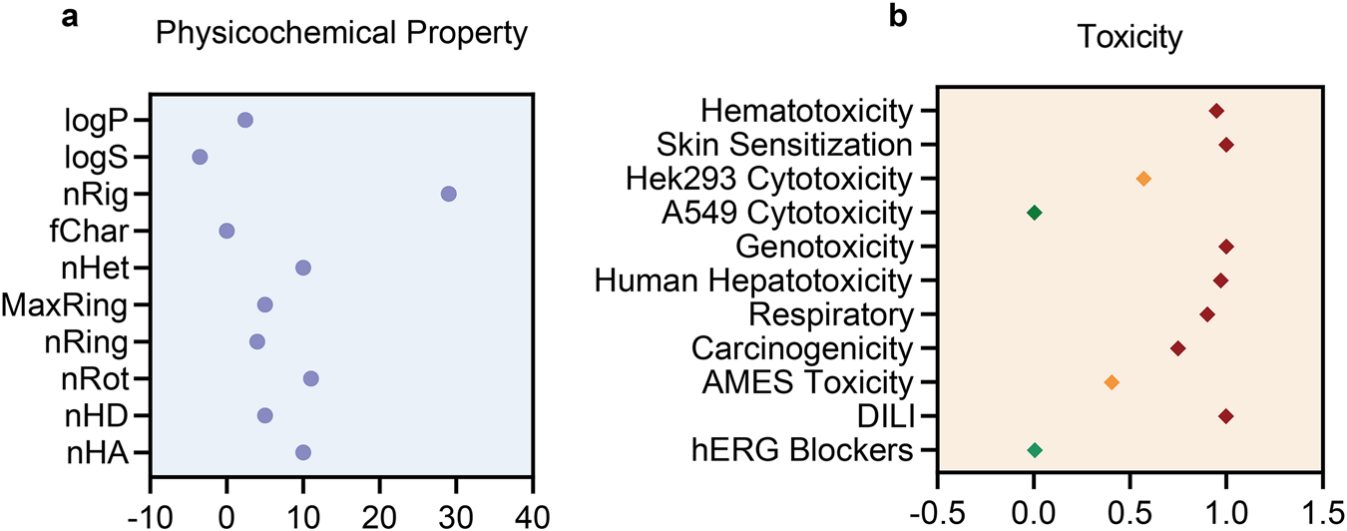
ADMET predictions. **a** Physicochemical Property of BV. **b** Toxicity of BV. Green, yellow, and red represented excellent, medium, and poor, respectively.

The physicochemical property analysis revealed a moderate lipophilicity (logP = 2.43), suggesting BV’s suitability for oral absorption (Fig. 8a). However, poor aqueous solubility (logS = −3.516) was also indicated. The high number of hydrogen bond acceptors and donors (nHA and nHD) might impair cellular permeability, while the elevated number of heteroatoms (nHet = 10.0) implied potential metabolic instability. Toxicity predictions demonstrated a high probability of drug-induced liver injury (DILI = 0.998), human hepatotoxicity (0.971), genotoxicity (1.0), and carcinogenicity (0.749), indicating significant hepatic toxicity, genotoxic risk, and carcinogenic potential (Fig. 8b). In contrast, Hek293 cytotoxicity (0.571) suggested moderate nephrotoxicity, while negligible cardiotoxicity (hERG blockers = 0.005) and pulmonary toxicity (A549 cytotoxicity = 0.004) were predicted. Assessment of inhibitory potential against seven major CYP450 isoforms yielded negative results for all enzymes. However, CYP2C9 and P-gp substrate assessment was positive (Table 2). The excretion results of BV indicated a short half-life and high plasma clearance (Supplementary Table 1).

**Table 2:** Results of the CYP450 enzyme inhibition prediction

CYP subtypes	Result
CYP1A2 inhibitor	−
CYP2C19 inhibitor	−
CYP2C9 inhibitor	−
CYP2C9 substrate	+
CYP2D6 inhibitor	−
CYP3A4 inhibitor	−
CYP2B6 inhibitor	−
CYP2C8 inhibitor	−
P-gp substrate	+
BBB permeant	−

## Discussion

The incidence of CDI has been increasing globally in recent years, especially with the emergence and prevalence of the hypervirulent strains^23^. Although antibiotics continue to be the first-line therapy for CDI, their disruption of gut microbiota remains a significant drawback, making them suboptimal for CDI treatments^2^. Bezlotoxumab, a human monoclonal antibody, treats CDI by inhibiting *C. difficile* TcdB’s ability to bind to host cells^24^. The monoclonal antibody actoxumab was also developed to treat CDI by neutralizing *C. difficile* TcdA^3^. While antibody stability influences efficacy in systemic therapy, it also becomes the determining factor for oral delivery due to GI tract challenges including acid-induced denaturation and protease degradation capabilities^25^. Small-molecule inhibitors targeting distinct domains in TcdA and TcdB have demonstrated significant potential in the treatment of CDI, offering a promising alternative to traditional therapies^16^. In our research, we employed an artificial intelligence assisted strategy to identify small natural molecules that target the structural domain of TcdB-DRBD. BV was identified as an effective, broad-spectrum natural agent with a strong binding capacity to all variants of TcdB.

Strains of *C. difficile* belonging to clade 2 can exclusively produce TcdB2 and TcdB4 toxins, which exhibit a remarkable protein sequence homology of 96.4%, highlighting their close evolutionary relationship^10^. Our previous study first demonstrated that TFPI was a colonic crypt receptor for TcdB from hypervirulent clade 2 *C. difficile*, and the active docking site was within the DRBD structural domain of TcdB2 and TcdB4^11^. The DRBDs of different TcdB variants recognized various receptors, and there were two receptor-binding epitopes (RBIs) on their surfaces, RBI-1 (residues 1430–1604) and RBI-2 (residues 563–621 and 1754–1850)^10^. RBI-1, located on the convex margin of the DRBD, binds to FZDs receptors (in TcdB1/3/5) or TFPI (in TcdB2/4), whereas RBI-2, a composite interface formed by the CPD, DRBD, and CROP domains, binds to CSPG4^26^. Except for the hypervirulent *C. difficile* clones producing TcdB2, it has been described that other clones such as sequence type 37^27^ and 81 (TcdB3)^28^, 11^29^ and 35 (TcdB1)^30^ led to severe CDI. Extensive molecular docking showed that BV can bind to RBI-1 of all TcdB variants. Interestingly, TcdB3, which was different from TcdB4 in the RBI typing, also showed a strong binding ability to BV, suggesting BV as a broad-spectrum therapeutic agent. BV also exhibited the potential to inhibit a wide range of TcdB toxicity, part of which has been demonstrated in our study. However, further studies should be carried out to confirm that BV possesses broad-spectrum activity against all remaining variants of TcdB in the near future.

BV, a non-toxic, green-hued, linear, water-insoluble pyrrole pigment, is a byproduct of heme oxygenase-1 (HO-1)^31^. Over the past few decades, BV has demonstrated beneficial effects in ischemia/reperfusion-related diseases^32^, inflammatory diseases^33^, graft-versus-host disease^34^, viral infections^35^, and cancer^36^. Unfortunately, BV was easily converted into bilirubin (BR) by the BV reductase ^37^, especially in mammals^38^. Furthermore, the transition of BV to BR sometimes made it difficult to distinguish between their two end products^39^. BV, administered intravenously and intraperitoneally, was rapidly reduced to Ursodeoxycholic Bile Acid (UCB) in vivo^38^. In the duodenum, BV was completely metabolized by intestinal bacteria, leading to the formation of bilirubin-10-sulfate (BRS)^40,41^. Therefore, the amount of recycled or converted BV should be further investigated. It has been shown that EVs possess some natural targeting properties due to their surface proteins and other components^42^, making them a promising natural nanocarrier^43^. Given that BV was unstable in the gut, this study used I-EVs to encapsulate BV with a pH-responsive (pH 7.4) controlled-release characteristic, thereby protecting BV before reaching the gut. Furthermore, after I-EVs were loaded with BV, the morphological change was observed in I-EV-BV, which may be due to the integration of BV molecules altering lipid arrangement and membrane curvature energy. It has been demonstrated that spherical vesicles with uniform curvature were more likely to interact with lipid rafts on the cell membrane, thereby promoting clathrin-mediated endocytosis^44^. Thus, it is reasonable to suggest that the spherical structure of the I-EV-BV may enhance cellular uptake efficiency. Zeta potential data also verified that the I-EV-BV enhanced structural stability as previously reported^45,46^. However, the exact amount of BV retained in the gut could not be determined after BV was released from I-EVs. As endogenous BV can be reduced to BR within minutes in mammals^40^ and the bioavailability of BV was relatively low as described in the studies^39^, we will further evaluate BR as a therapeutic agent in vitro to investigate its efficacy in inhibiting TcdB2 activities. In addition, endogenous BV might be present in extremely low concentrations in the body. Therefore, it was considered negligible in our study. However, it is still unknown on what other potential alterations of BV might occur through the intestinal tract, via the gut bacteria, and by enterohepatic circulation. The effects of bile acids on the composition of administered BV are also unknown. Both of which are indeed worthy of being further studied through interaction between BV and the gut bacteria, and other experiments. Moreover, the cost of high-purified BV is high, and the main source of commercial BV currently is the oxidation of BR^47^, reducing the generalizability of BV. Additionally, the current extraction methods are not satisfactory to produce sufficient purified BV, which greatly hindered the broad utilizations of BV^48^. So far, there have still been no reported technologies for BV extraction from fecal samples, maybe due to its enzymatic degradation during sample processing, resulting in minimal residual BV in feces. Furthermore, clinical data on the pharmacokinetics of BV are still limited ^39^. ADMET predictions indicated negative results for all seven CYP450 isoenzyme inhibitors, suggesting that BV is safe to be co-administered without significant drug-drug interaction risks^49^. However, the assessment result also indicated BV’s predominant metabolic reliance on CYP2C9 substrate. Despite these findings, the challenges of poor solubility and metabolic instability, as indicated by physicochemical properties, remain to be addressed. Thus, we are going to modify and optimize the BV structure to improve its stability, making it more suitable for disease treatment.

Microbiota plays an important role in protecting the host from pathogen invasion^50^*. Firmicutes* are the main bacterial gateway to the intestinal tract in healthy humans^51^. Previous studies have found that *Firmicutes* released cell wall glycoconjugates that induced IL-34 production, thereby initiating host immune defense against systemic inflammation^52^, enhancing lung antimicrobial capacity^53^ and antiviral immunity^54^, and increasing the host’s resistance to sepsis^55^. IL-34, on the other hand, can coordinate inflammatory and immune responses by signaling the nuclear factor-κB (NF-κB) pathway^56^. It was found that CDI is usually accompanied by two innate immune responses, leading to the activation of inflammatory vesicles and the production of proinflammatory cytokines, including IL-1β andCXCL1, and thereby activating the NF-κB pathway^57^. Our study found that BV treatment is associated with an increase in the genus *Firmicutes*. Thus, we speculate that BV could modulate the immune homeostasis of CDI gerbils by increasing the number of post-mitotic bacilli through IL-34, which needs further investigation. KEGG pathway enrichment analysis also revealed that only three metabolic pathways differed significantly between the untreated and BV-treated groups without CDI. Conversely, CDI resulted in 52 differentially enriched pathways compared to CDI treated by BV alone, highlighting infection-specific perturbation in molecular networks. These results suggest that the CDI group might exhibit higher levels of metabolic activity, energy demand, and cellular stress, possibly associated with higher oxidative stress, inflammatory response, and apoptosis than those in the BV treated group. However, the BV treated group exhibited lower metabolic activity and energy demand, perhaps associated with a more stable cellular state and lower levels of stress, elucidating the key role of BV in metabolic regulation and disease development, as well as providing new perspectives for understanding the process of BV treating CDI.

SCFAs are the main products of microbial fermentation in the gut, particularly acetic acid, propionic acid, and butyric acid^58,59^. They contribute to the maintenance of intestinal homeostasis and regulation of energy metabolism^60^. This study found that BV exerted anti-CDI effects by reconstructing the intestinal SCFAs metabolic network. After BV treatment, the levels of acetic acid, propionic acid, and butyric acid in the gerbil’s intestinal contents increased significantly. SCFAs have been reported to inhibit the production of *C. difficile* toxins^61^. Among these SCFAs, butyric acid has been the most extensively studied. In vivo studies demonstrated that butyric acid activates the HIF-1 pathway to protect mice from CDI^62^ and also ameliorates CDI by promoting secondary bile acid metabolism^63^. Moreover, in vitro evidence further confirmed that butyric acid directly inhibits the growth of *C. difficile*^64^. Meanwhile, acetic acid and propionic acid contributed to anti-inflammatory processes by modulating leukocyte function^65,66^. Our study also observed significant alleviation of CDI symptoms and reduction in expression levels of proinflammatory cytokines following BV treatment, indicating that BV not only exerts its therapeutic effects against CDI but also moderates SCFAs levels in the gut, which might present a synergistic effect on CDI treatment.

Notably, there are many butyric acid-producing bacterial genera in the *Firmicutes* phylum, like *Ruminococcaceae* and *Roseburia*^67^. Compared to the CDI group, BV treatment significantly increased the abundance of *Ruminococcaceae*, a major producer of butyric acid^68^.This microbial shift provided a plausible explanation for the elevated butyric acid levels observed following BV intervention. *Roseburia* was also considered a beneficial gut bacterium^69^, which contributes to intestinal homeostasis by maintaining the Treg/Th17 balance, thereby protecting the intestinal epithelial barrier and alleviating colitis symptoms^70,71^. Furthermore, the proliferation of *Firmicutes* may competitively deplete intestinal carbon sources (e.g., succinate and N-acetylglucosamine) that are essential for *C. difficile* growth^72^. Therefore, it might be reasonable that the recovery of *Firmicutes* after BV treatment led to the inhibition of *C. difficile* growth. However, it remains to be elucidated why BV induced *Firmicutes* enrichment.

This study had some limitations. Primarily, we focused on BV’s interaction with the DRBD domain of TcdB variants, the capacity of BV to mitigate the impact of other critical toxins, TcdA and CDTs, in CDI were not investigated. Secondly, only one hypervirulent strain was used to evaluate the performance of BV in our study, and other genotypes were not included. Our study has demonstrated that all the TcdB variants interacted with BV. TcdB1-4, which induced cytotoxicity of CaCo-2 cells, was inhibited by BV. Our previous study also illustrated that most of genotypes expressed TcdB1-4^10^. Therefore, we will further evaluate the capacity of BV to inhibit TcdB5-8, induce cytotoxicity and alleviate CDI caused by other genotype strains such as RT078, RT014 and RT106 is needed. Thirdly, recent findings suggested that TcdB-induced cell death is dependent on reactive oxygen species (ROS)^68^. In light of this finding, it remains unclear whether BV can protect against ROS through its antioxidant properties during CDI, a question that warrants further investigation. Lastly, the sample size in the CDI gerbil model was relatively small, which possible lead to the no significant differences in the survival rate among all the groups. We will increase the sample size of gerbils and collect intestinal contents at longitudinal multiple time points, ranging from less than 24 h to 96 h, and even longer, to further evaluate in vivo the capacity of BV to treat CDI, measuring the survival rate, dynamic change of gut microbiota and TcdB clearance kinetics in long-term infection. Furthermore, we should also highlight the prolonged incubation that leads to secondary effects like small-molecule degradation or false resistance.

In conclusion, we have successfully identified a natural small-molecule inhibitor, BV, that exhibits broad interaction with all TcdB variants. Notably, BV, which is non-cytotoxic, did not affect the growth of *C. difficile* cells but effectively inhibited TcdB1-4 induced toxicity. I-EV-BV, characterized by its pH-responsive controlled-release feature, not only effectively alleviated the symptoms of CDI and improved survival rates, but also restored beneficial intestinal bacteria such as *Firmicutes*. Additionally, BV also exerted its anti-CDI effect by rebuilding the SCFAs metabolic network, leading to an increase of acetic acid, propionic acid, and butyric acid. Therefore, our study highlighted BV, for the first time in this field of research, as a promising non-antibiotic, natural, small-molecule therapeutic agent for attenuating TcdB-induced pathological injuries and ultimately treating CDI.

## Methods

### Screening small molecular candidates and molecular docking

The three-dimensional protein structure of *C. difficile* TcdB4 (PDB ID: 7V1N) was downloaded from the RCSB protein database^11^, and the structures of TcdB1-3 and 5–8 clades were predicted using Alphafold3^73^, which were used for subsequent docking. The structure of small molecular was obtained from DrugBank in SDF format^74^. The molecular docking process was conducted using AutoDock Vina (v1.2.4)^75^. The docking parameters are shown in Supplementary Table 2. Binding affinity was automatically calculated at the end of the process, and optimal binding modes were saved for each ligand. PyMOL was then employed to visualize polar contacts between molecules and amino acid residues^76^. The Schrödinger 2D Sketcher module was utilized to visualize non-covalent interactions.

### Molecular dynamics simulations

The all-atom MD simulations were conducted using the GROMACS 2025.1 package^77^ to investigate the stability and binding interactions of the ligand-TcdB4 complexes. System was constructed based on the TcdB4 (PDB ID: 7V1N) structure and ligand poses derived from AutoDock Vina, with topology files generated for the protein using the AMBER99SB-ILDN force field^78^ and ligands via the CGenFF server. Systems were solvated in a TIP3P water box^79^ extending 1.0 nm from the solute, neutralized with Na⁺/Cl⁻ ions, and energy-minimized using the steepest descent algorithm (500 steps) to eliminate steric clashes (Supplementary Data 2). Sequential equilibration was performed under 100 ps, 300 K, V-rescale thermostat NVT and 100 ps, 1 bar, Parrinello-Rahman barostat NPT ensembles to stabilize temperature and pressure. Production simulations were executed for 100 ns with a 2 fs integration timestep, employing LINCS constraints for bond lengths and periodic boundary conditions. Trajectory frames were saved every 10 ps for subsequent analysis. Structural stability was assessed via RMSD of protein backbone and ligand, while residue flexibility was quantified through RMSF. Binding free energies decomposition were computed via the gmx_MMPBSA tool^80^ to evaluate van der Waals, electrostatic, and solvation contributions. Simulations were independently performed with different initial velocity distributions and random seeds three times. The results from these simulations with no significant differences were considered valid, indicating that the final outcomes were robust and independent of the initial configuration. The timescale of the events under investigation, such as the binding and conformational changes of BV with TcdB4, was within the scope of the current MD simulation timescale. The initial coordinate file, simulation input file, and output coordinate file are provided in the Supplementary Data 3.

### UV absorption spectra

The purified TcdB2 was prepared in our laboratory^81^. The BV (Santa Cruz, 55482-27-4, USA) was dissolved in DMSO, and TcdB2 was dissolved in PBS buffer at final concentrations of 30 μM and 1 μM, respectively. Subsequently, 500 μL of BV and 500 μL of TcdB2 were well mixed at room temperature for 1 h. The UV-visible absorption spectra of the BV, the TcdB2, and the mixture of BV and TcdB2 were scanned with a UV spectrophotometer (Shimadzu, UV-2600, Japan) in the wavelength range from 250 to 450 nm.

### Circular dichroism

Circular dichroism was performed using a circular dichroism spectrometer (Jasco J-1500, Japan) equipped with a Peltier temperature control system, covering a wavelength range of 180–700 nm. A mixture of 2 mg/mL TcdB2 and 30 μM BV was incubated at room temperature for 30 min in darkness. The solutions of TcdB (2 mg/mL), BV (30 μM), and TcdB+BV were sequentially scanned using a 1 mm quartz cuvette (Hellma, Germany), with a data pitch (ΔX) of 0.1 nm and a scanning speed of 100 nm/min at 20 °C. Nitrogen purging was applied to minimize oxygen absorption interference in the far-UV region. The CD spectra were calibrated using solvent baselines.

### Surface plasmon resonance (SPR)

The interaction between BV and TcdB2 was determined using a Biacore (1 K, Cytiva, USA) biomolecular interaction analyzer. TcdB2 proteins were diluted to 50 μg/mL with sodium acetate and immobilized in 2 channels of the chip at a flow rate of 10 μL/min to generate a coupling map. BV was twofold diluted to a series of concentrations in a 96-well plate and coupled to TcdB2 by passing through the chip from a low concentration to a high concentration. After each concentration point flowed, the chip was regenerated with 10 mM glycine hydrochloride (pH 2.0) solution for 5 min, and the process was repeated until the corresponding concentrations of all compounds had been run. Binding and dissociation constants were obtained by fitting the data globally to a ratio of 1:1 Langmuir binding model using the Biacore Insight evaluation software (Cytiva, Marlborough, MA, USA)^82^. The interaction analyzer recorded the response units (RU) of the sample points on the chip surface. Vancomycin and the E3 neutralization antibody^83^ prepared in our laboratory were used as negative and positive controls, respectively.

### BV affecting *C. difficile* growth

The *C. difficile* strain (ATCC BAA-1870, ST1/RT027) was cultured in Brain Heart Infusion broth containing different concentrations of BV and vancomycin at 37 °C, respectively. The values at OD_600nm_ were measured at different time points and growth curves were plotted. Meanwhile, *C. difficile* was grown on a Bruce agar plate containing different concentrations of BV and vancomycin, respectively, and incubated at 37 °C for 48 h in an anaerobic chamber with GENbag Anaer (BioMérieux, Marcy l’Etoile, France) according to the CLSI (M100 ED34-2024). Vancomycin was used as a positive control.

### BV’s toxicity and protective effect against TcdB

CaCo-2 cells were cultured in Minimum Essential Medium (Gibco, USA) containing 20% (v/v) fetal bovine serum (Biological Industries, Israel), 1% (v/v) penicillin/streptomycin and 1% (v/v) non-essential amino acids (Gibco, USA). Following the seeding of CaCo-2 cells at a density of 5 × 10^4^ cells per well in a 96-well plate and incubation at 37 °C with 5% CO^2^ for 48 h, different concentrations of BV were introduced into the wells at intervals of 24, 48, and 72 h. Cell proliferation was measured using the CCK-8 assay^84^. To ascertain the protective effect of BV against TcdB1-4 toxicity, CaCo-2 cells with a density of 5 × 10^4^ cells per well were cultured in a 96-well plate at 37 °C with 5% CO^2^ for 48 h. Then, 1 pM of each TcdB variant (types 1, 3, and 4, gifts from Westlake University) was added to the cells with or without 50 μM BV. After incubating for 24 h, the CaCo-2 cell morphology was observed using microscopy (Olympus, CKX53, Japan). The neutralization antibody E3 prepared in our laboratory was used in parallel to confirm TcdB induced cell rounding as a positive control. Cell viability was quantitatively determined using the Cell Counting Kit-8 (CCK-8) assay. Briefly, CaCo-2 cells were seeded at a density of 1 × 10⁶ cells per well in a 24-well plate and subjected to identical stimulation protocols for 1 h. Supernatants were subsequently harvested for detection of TcdB via an immunoassay as described below.

### An immunoassay for the detection of TcdB toxin

The excised intestinal contents were soaked in PBS overnight. Then 100 µL of the soaked supernatant and the purified TcdB2 were added to the plate and incubated overnight at 4 °C. The plate was washed three times with PBS, incubated with blocking solution (PBST + 0.5% BSA) for 2 h, washed three times with PBST, incubated with primary antibody E3 (1:1000) for 2 h, and washed four times with PBST. The secondary antibody Myc (1:5000) (Abcam, UK) was incubated for 1 h using the same washing method as described above. TMB chromogen solution (Beyotime, China) for ELISA was added and incubated for 15 min in the dark. Finally stop solution for TMB substrate (Beyotime, China) was added, and the OD value was measured using the microplate reader (Molecular Devices, SpectraMax M5E, San Jose, CA, USA) at 450 nm.

### Isolation, identification, and characteristics of I-EVs from intestinal epithelial cells

The EV isolation method utilized in this study adhered to the standard indications of the International Society for EVs^85^. C57BL/6 mice were purchased from the Animal Center of Hangzhou Medical College, and the experimental animal license number was SCXK (Zhe) 2019-0002. All animal procedures were conducted in accordance with the Guidelines for Care and Use of Laboratory Animals of Zhejiang Institute for Food and Drug Control and were approved by the Animal Ethics Committee of Zhejiang Institute for Food and Drug Control. I-EVs were obtained from the colorectal epithelium of adult male C57BL/6 mice aged 8 weeks. The mouse’s large intestine was surgically extracted and ground in a sufficient amount of cold PBS. It was then digested with 1 mg/mL type II collagenase (Gibco, USA) for 2 h at 37 °C. I-EVs were extracted as previously reported^86^. The harvested I-EVs were resuspended in PBS. BCA was used to detect the concentration of I-EVs (ThermoFisher, Waltham, MA, USA). The prepared I-EVs were examined using a transmission electron microscope (Hitachi HT7700, Japan), and then tested for particle NTA and Zeta potential using the Zeta VIEW instrument (Particle Metrix, Germany). And 50 µg of total proteins extracted from I-EVs were prepared for western blotting. TSG101^86^(Abcam 125011), CD63^86^(Abcam 213090), ALIX^87^(Abcam 275377), A33^86^(Cell Signaling Technology, #2449, Danvers, MA, USA), and Calnexin^87^(GT1563) as I-EVs’ markers were detected by chemiluminescence using the immobilon western HRP substrate (Merck Millipore, MA, USA). Protein preparation from intestinal epithelial cells (whole extract) was used as a negative control.

### Preparation of I-EV-BV and in vitro release

I-EVs (50 µg) mixed with BV (50 µg) were sonicated using an ultrasonic cell crusher (Scientz, China) for 30 min and incubated at 37 °C for 60 min to facilitate restoration of the I-EVs’ membrane. The prepared I-EV-BV was combined with 2 mL of buffers with pH 2 (Aladin, China), pH 5 (Aladin, China), and pH 7.4 (BBI, China), respectively and placed in pre-treated dialysis bags (MW:12,000 -14,000). The bags were then incubated in a shaking incubator at 37 °C. 1 mL of dialysate was removed at 0.5, 1, 2, 4, 6, 8, 12, 24, 36, and 48 h and supplemented with 1 mL of buffer. The absorbance value of each dialysate sample was determined by UV spectrophotometry at the maximum wavelength and substituted into the standard curve equation to derive the concentration of the corresponding sample. The prepared I-EV-BV was centrifuged at 12,000 × *g* for 15 min. The supernatant was then collected, and the drug load (DL) was calculated as described above. The I-EVs and I-EV-BV were incubated with DiI dye (Beyotime, China) in the dark at 37 °C for 1 h, then centrifuged at 50,000 × *g* for 30 min to remove excess dye, and then stored in the dark for further analysis on biological distribution in vivo.

### Establishment of a Mongolian gerbil model for CDI

The primary CDI model was established according to our previous report 18. Mongolian gerbils were purchased from the Animal Center of Hangzhou Medical College, and the experimental animal license number was SCXK (Zhe) 2019-0002. All animal procedures were conducted in accordance with the Guidelines for Care and Use of Laboratory Animals of Zhejiang Institute for Food and Drug Control and were approved by the Animal Ethics Committee of Zhejiang Institute for Food and Drug Control. These male gerbils (8-week-old) were housed in Zhejiang Institute for Food and Drug Control (23 °C ± 2 °C room temperature, 55% ± 5% relative humidity and 12 L:12D photoperiod) with free access to water and food. A total of 40 animals were randomly divided into six groups: Untreated, vancomycin (50 mg/kg), BV (35 mg/kg), I-EV-BV (35 mg/kg), I-EVs (100 μg/animal), and CDI (PBS). The gerbils were monitored daily for changes in body weight, the presence of wet tails, arched backs, depression and the development of disease symptoms. DAI was scored according to the assay with minor modification ^88^. Score 0: normal body weight with formed stools. Score 1: 1–5% weight loss with soft stools. Score 2: 5–10% weight loss with mucoid stools. Score 3: 10–20% weight loss with watery stools. After the gerbils were sacrificed, the intestinal tissues and intestinal contents from the different groups were collected and prepared for H&E staining and detection of TcdB concentration. We have complied with all relevant ethical regulations for animal use.

### Biological distribution in vivo

To investigate the biological distribution of I-EVs, DiI-labeled I-EV-BV and I-EVs were orally administered to mice via gavage, respectively. After euthanasia, organs including the heart, liver, spleen, lungs, kidneys, and intestines were surgically excised. Fluorescence imaging of organ-specific I-EVs distribution was subsequently captured using a multimodal in vivo imaging system (BLT, AniView100, China).

### Elisa

The concentrations of pro-inflammatory cytokines TNF-α and IL-6 in gerbil’s colon tissues were quantified using the LEGEND MAX™ Mouse TNF-α and IL-6 ELISA Kits (Bio Legend, USA) according to the manufacturer’s instructions.

### Counting of *C. difficile* strains from intestinal contents

*C. difficile* strains were isolated from gerbil’s intestinal contents according to the assay with minor modifications^89^. The 100 mg of intestinal contents collected from gerbils were added to 800 μL of alcohol and mixed well at room temperature. The mixture was centrifuged at 8000 rpm for 5 min, and the supernatant was discarded. The pellet was resuspended in saline solution and then inoculated onto Cefoxitin-Cycloserine Fructose Agar plates (Oxoid, Basingstoke, UK) for incubation anaerobically at 37 °C for 48 h. The colony-forming units were counted.

### Microbiota analysis

Mongolian gerbils (male, 8-week-old) were divided into four groups and treated orally with vancomycin (50 mg/kg), BV (35 mg/kg), fidaxomicin (30 mg/kg) and drinking water without any additional drugs for 3 days. Intestinal contents were collected for analysis. The Mongolian gerbils infected with *C. difficile* (ATCC BAA-1870) were treated as in our previous report^18^. After 3 days of BV treatment, the intestinal contents were collected for 16S rDNA amplicon sequencing. Genomic DNAs of the intestinal contents were extracted using the PowerSoil^®^ DNA Isolation Kit (MO BIO, Cat. No. 12888). Sequencing libraries were generated using the TruSeq DNA PCR-Free Sample Preparation Kit (Illumina, San Diego, CA, USA) and sequenced using the NextSeq 2000 platform (Illumina, San Diego, CA, USA). To study the species composition diversity of microbiota, the effective tags of all the samples were clustered using UPARSE. The sequences were clustered into Operational Taxonomic Units (OTUs), and then the representative sequences of OTUs were annotated with species. Differences in species diversity among the groups were analyzed by exploiting the Shannon and ACE indices. A comparative analysis among the groups was conducted using NMDS (Non-Metric Multi-Dimensional Scaling) and a heatmap based on the OTU level. Furthermore, Tax4Fun (v0.94)^90^ was employed to predict the differential abundance of KEGG pathways.

### SCFAs quantitative analysis

SCFAs levels were measured using UPLC-ESI-MS/MS (AB Sciex, Qtrap 5500, USA). The 100 mg of intestinal contents were mixed with two small steel beads and 300 μL of acetonitrile solution, and the mixture was then placed in a grinding machine for homogenization (45 Hz, 2 min) (Wonbio-E, China). The mixture was sonicated in an ice-water bath for 10 min, followed by centrifugation for 10 min (4 °C, 12,000 rpm) (BIORIDGE, TGL-16MS, China). The supernatant was diluted 5-fold with acetonitrile. The 80 μL of diluted supernatant was mixed with 40 μL of 200 mM 3-NPH (prepared in 50% acetonitrile-water, v/v) and 40 μL of 120 mM EDC containing 6% pyridine (prepared in 50% acetonitrile-water, v/v). The mixture was then incubated at 40 °C for 30 min. The components in the supernatant were separated using an ACQUITY UPLC BEH C18 column (Waters, USA) (100 × 2.1 mm, 1.7 μm), with mobile phases A (0.1% formic acid in water) and B (acetonitrile/methanol = 2:1). The flow rate was maintained at 0.35 mL/min throughout the analysis.

### ADMET assessment

The ADMET assessment was performed including physicochemical properties, toxicity, metabolism and excretion of BV by ADMETlab 3.0^91^ and SwissADME^92^.

### Statistics and reproducibility

All data were statistically analyzed using GraphPad Prism 10^93^. Error bars represented analyses based on multiple biological replicates. All boxplot whiskers extend to the highest and lowest values and the middle line indicates the median. Normality was assessed using the Shapiro-Wilk test, and homogeneity of variances was evaluated with *F* tests. The data are presented as means ± SD. Data analysis was performed using the One-way ANOVA or Two-way ANOVA with Tukey’s multiple comparisons test or Dunnett’s multiple comparisons test, and *p* < 0.05 was considered statistically significant. For each experiment, the number of replicates and sample sizes were noted and explained in each figure legend.

### Reporting summary

Further information on research design is available in the Nature Portfolio Reporting Summary linked to this article.

## Supplementary information

Supplementary Information

Description of Additional Supplementary files

Supplementary data 1

Supplementary data 2

Supplementary data 3

Supplementary data 4

Reporting Summary

## Data Availability

Raw 16S rRNA sequencing data have been deposited in the SRA database of NCBI under accession numbers PRJNA1331431. All data supporting the findings of this study are available within the paper and its Supplementary Information and Supplementary files. The Uncropped and unedited blot/gel images are available in the Supplementary Information. The numerical source data for graphs and charts are provided in the Supplementary Data 4. Any other data supporting the findings of this study are available from the corresponding author upon reasonable request.
