# Supplementary material for: Biliverdin targeting TcdB-DRBD inhibits Clostridioides difficile virulence and restores gut microbiota in Mongolian gerbils (Meriones unguiculatus)
Source: Commun Biol. 2025 Nov 25;8:1663. doi: 10.1038/s42003-025-09059-8 (PMC12647718; doi:10.1038/s42003-025-09059-8)
Supplement: Supplementary file 1 — Supplementary Information [file 42003_2025_9059_MOESM1_ESM.pdf]

Supplementary Information

Biliverdin targeting TcdB-DRBD inhibits *Clostridioides difficile* virulence and restores gut microbiota in Mongolian gerbils (Meriones unguiculatus)

*Shuangshuang Wan[1,2,3], Yu Lei[1], Yue Jin[1], Runze Wang[1], Meng Zhang[1], Qikai Shi[1], Hui Hu[1,2,3], Yulei Tai[1,2,3], Yun Luo[4], Zheng Xu[1,2,3], Rong Kuang[5], Xiaojun Song[3], Yu Chen[1,2,3], Dazhi Jin[1,2,3]**

Supplementary Information includes:

Supplementary Figures. 1-4

Supplementary Tables. 1-2

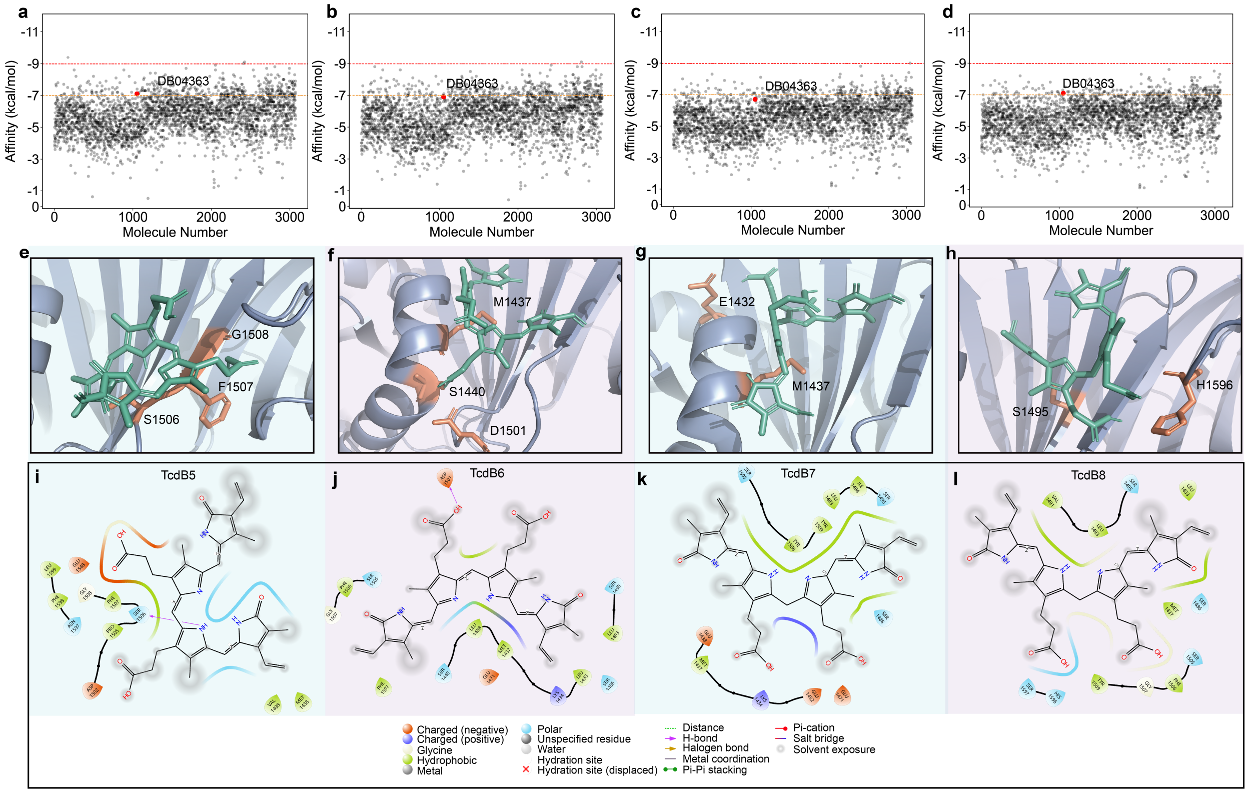

Supplementary Figure. 1. Results of screening and molecular docking. Scatter plots with binding energies of small molecule compounds screened against TcdB5-8 (**a-d**). BV and TcdB5-8 complex structures for AutoDock Vina simulations (**e-h**). BV is shown in green, and key amino acid residues are in yellow. The non-covalent interaction between BV and TcdB5-8 (**i-l**) residues at the molecular level.

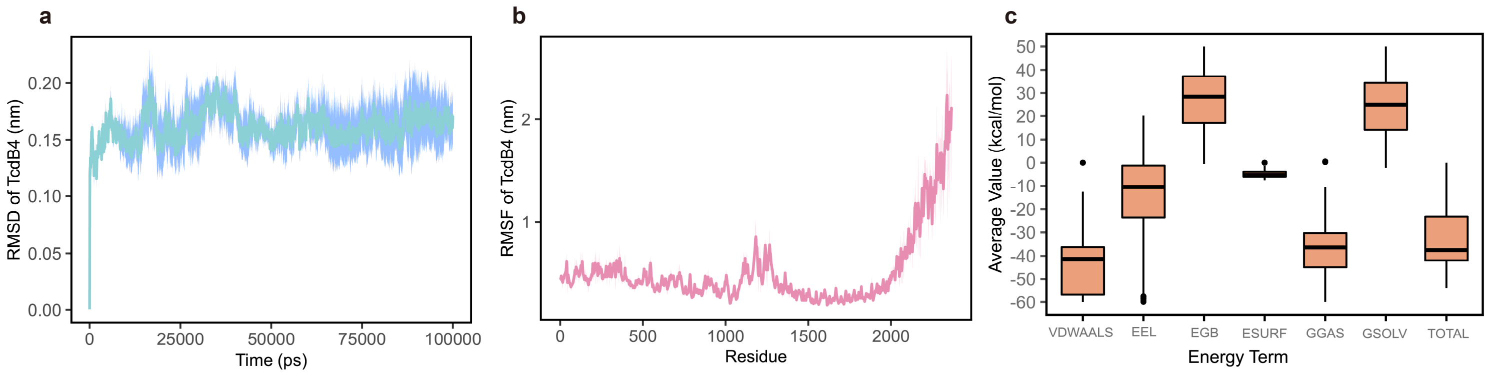

Supplementary Figure. 2. GROMACS was used to perform molecular dynamics simulations. a. RMSD of molecular dynamics simulations of the TcdB4-BV interaction. (mean + 95 % CI, n= 3) Blue indicated the confidence interval. **b.** RMSF of molecular dynamics simulations of the TcdB4-BV interaction (mean + 95 % CI, n= 3). **c.** Energy decomposition analysis of TcdB4 binding to BV calculated using the MM/PBSA method (n=5000).

Supplementary Figure. 3. The uncropped western blot results.

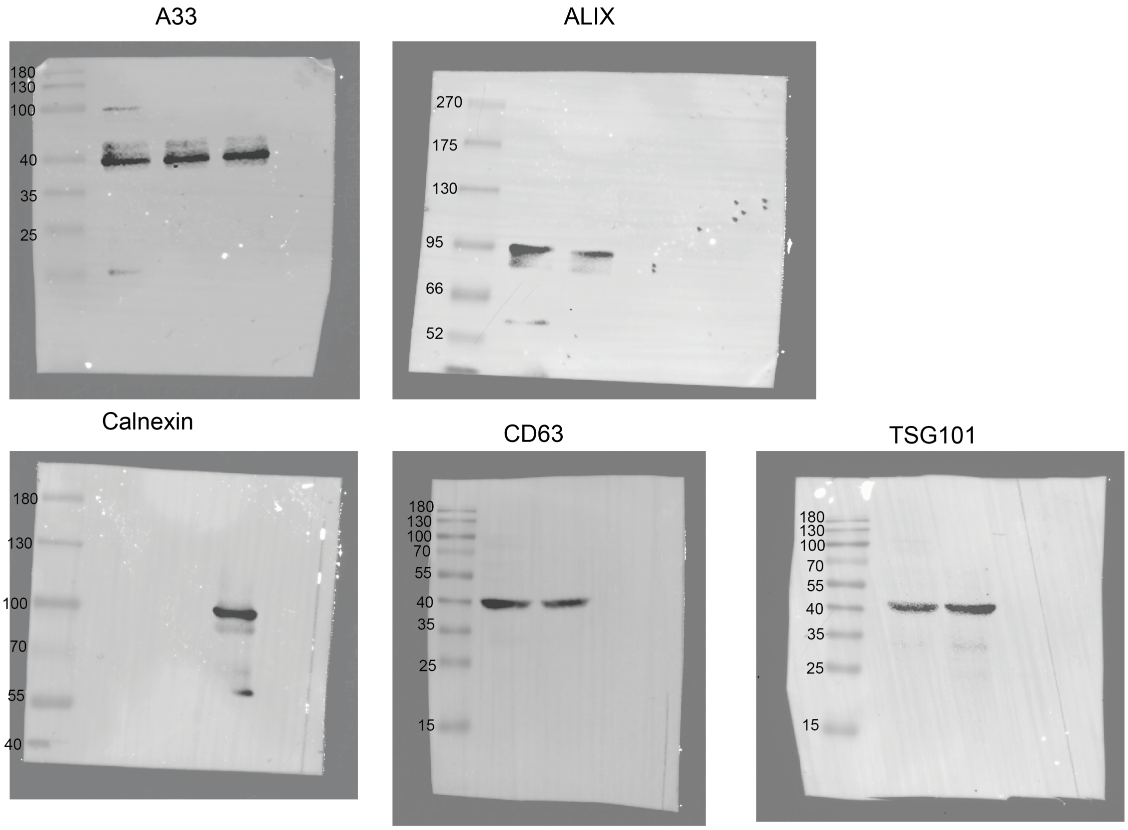

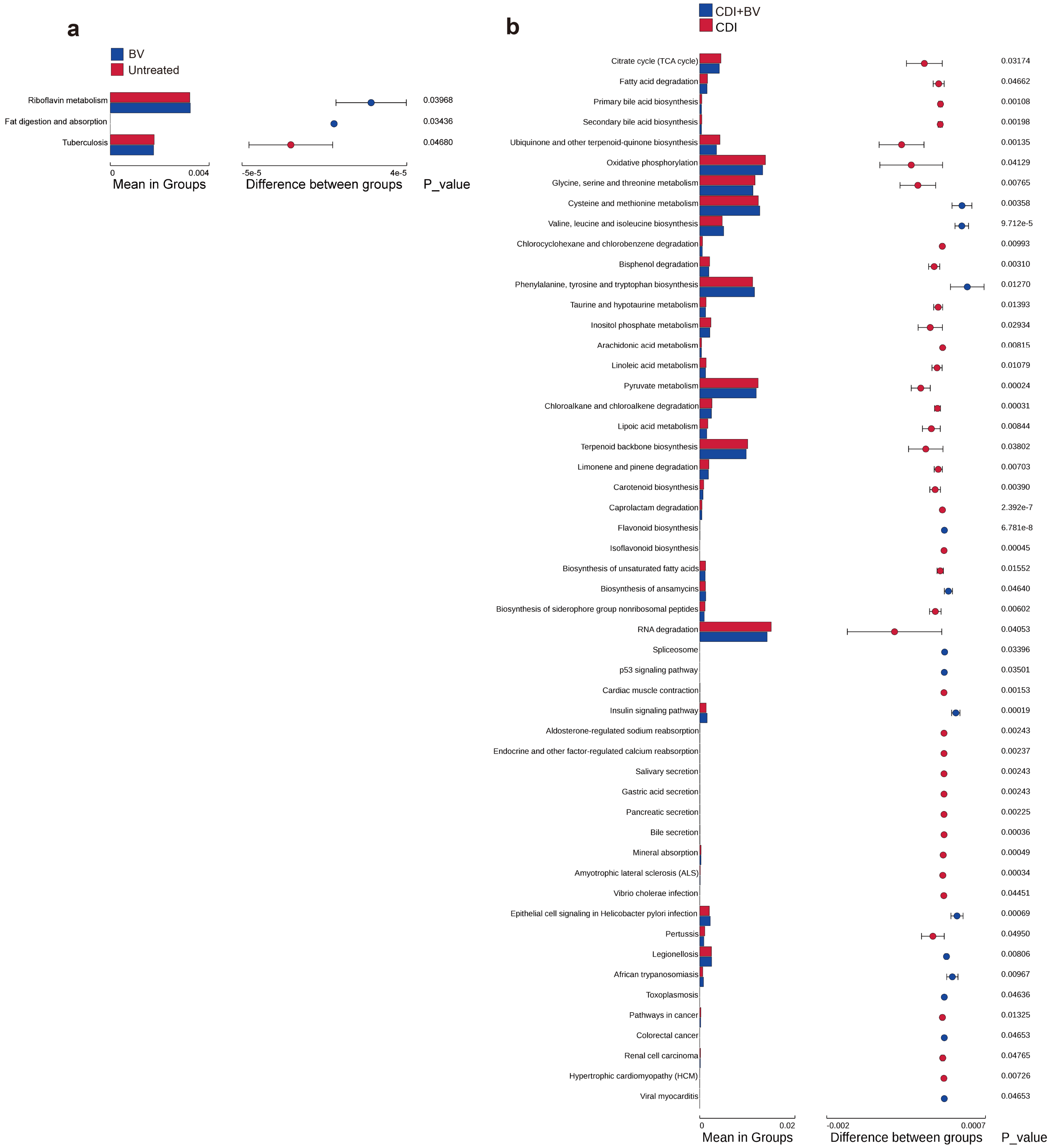

Supplementary Figure. 4. KEGG pathway enrichment analysis for the gut microbiota of different groups. a. BV treated group versus untreated group. **b.** CDI group versus BV treated group.

Supplementary Table 1. Results of the predicted BV excretion

Excretion	Result
CL_{plasma}	1.281 mL/min/kg
$T_{1/2}$	1.48 h

Supplementary Table 2. AutoDock Vina molecular docking parameters

Category	Attributes	Parameters	Brief description
Search space	Center x	221.8	X coordinate of the center
	Center y	157.5	Y coordinate of the center
	Center z	138.5	Z coordinate of the center
	Size x	20	Size in the X dimension (Ångstrom)
	Size y	20	Size in the Y dimension (Ångstrom)
	Size z	20	Size in the Z dimension (Ångstrom)
Miscellanea	Num modes	9	Maximum number of binding modes to generate
	Energy range	10	Maximum energy difference between the best binding mode and the worst one displayed (kcal/mol)
