# Supplementary material for: Biliverdin targeting TcdB-DRBD inhibits Clostridioides difficile virulence and restores gut microbiota in Mongolian gerbils (Meriones unguiculatus)
Source: Commun Biol. 2025 Nov 25;8:1663. doi: 10.1038/s42003-025-09059-8 (PMC12647718; doi:10.1038/s42003-025-09059-8)
Supplement: Supplementary file 2 — Description of Additional Supplementary files [file 42003_2025_9059_MOESM2_ESM.docx]

Description of Additional Supplementary Files

File name: Supplementary data 1

Description: Virtual screening results of AutoDock Vina

File name: Supplementary data 2

Description: System setup for molecular dynamics simulations

File name: Supplementary data 3

Description: Files containing input coordinates, input files, and output coordinates

File name: Supplementary data 4

Description: The source data behind the graphs in the paper
