# Supplementary material for: Biliverdin targeting TcdB-DRBD inhibits Clostridioides difficile virulence and restores gut microbiota in Mongolian gerbils (Meriones unguiculatus)
Source: Commun Biol. 2025 Nov 25;8:1663. doi: 10.1038/s42003-025-09059-8 (PMC12647718; doi:10.1038/s42003-025-09059-8)
Supplement: Supplementary file 7 — Reporting Summary [file 42003_2025_9059_MOESM7_ESM.pdf]

Corresponding author(s): Dazhi Jin

Last updated by author(s): 2025-9-11

Reporting Summary

Nature Portfolio wishes to improve the reproducibility of the work that we publish. This form provides structure for consistency and transparency in reporting. For further information on Nature Portfolio policies, see our Editorial Policies and the Editorial Policy Checklist.

Statistics

For all statistical analyses, confirm that the following items are present in the figure legend, table legend, main text, or Methods section.

n/a	Confirmed	
☐	☒	The exact sample size (*n*) for each experimental group/condition, given as a discrete number and unit of measurement
☐	☒	A statement on whether measurements were taken from distinct samples or whether the same sample was measured repeatedly
☐	☒	The statistical test(s) used AND whether they are one- or two-sided<br>*Only common tests should be described solely by name; describe more complex techniques in the Methods section.*
☒	☐	A description of all covariates tested
☐	☒	A description of any assumptions or corrections, such as tests of normality and adjustment for multiple comparisons
☐	☒	A full description of the statistical parameters including central tendency (e.g. means) or other basic estimates (e.g. regression coefficient) AND variation (e.g. standard deviation) or associated estimates of uncertainty (e.g. confidence intervals)
☐	☒	For null hypothesis testing, the test statistic (e.g. *F*, *t*, *r*) with confidence intervals, effect sizes, degrees of freedom and *P* value noted<br>*Give P values as exact values whenever suitable.*
☒	☐	For Bayesian analysis, information on the choice of priors and Markov chain Monte Carlo settings
☒	☐	For hierarchical and complex designs, identification of the appropriate level for tests and full reporting of outcomes
☒	☐	Estimates of effect sizes (e.g. Cohen's *d*, Pearson's *r*), indicating how they were calculated

Our web collection on statistics for biologists contains articles on many of the points above.

Software and code

Policy information about availability of computer code

Data collection	No special softwares were used
Data analysis	We used GraphPad Prism version 10, Adobe Illustrator to analyse the data in this study.

For manuscripts utilizing custom algorithms or software that are central to the research but not yet described in published literature, software must be made available to editors and reviewers. We strongly encourage code deposition in a community repository (e.g. GitHub). See the Nature Portfolio guidelines for submitting code & software for further information.

Data

Policy information about availability of data

All manuscripts must include a data availability statement. This statement should provide the following information, where applicable:

- Accession codes, unique identifiers, or web links for publicly available datasets
- A description of any restrictions on data availability
- For clinical datasets or third party data, please ensure that the statement adheres to our policy

The 16S rRNA sequencing data generated in this study have been deposited in the CNSA under accession numbers CNP0007998 and CNP0007999.The numerical source data for graphs and charts are provided in the Supplementary Data 2. Any other data supporting the findings of this study are available from the corresponding author upon reasonable request.

Research involving human participants, their data, or biological material

Policy information about studies with human participants or human data. See also policy information about sex, gender (identity/presentation), and sexual orientation and race, ethnicity and racism.

Reporting on sex and gender	N/A
Reporting on race, ethnicity, or other socially relevant groupings	N/A
Population characteristics	N/A
Recruitment	N/A
Ethics oversight	N/A

Note that full information on the approval of the study protocol must also be provided in the manuscript.

Field-specific reporting

Please select the one below that is the best fit for your research. If you are not sure, read the appropriate sections before making your selection.

☒ Life sciences ☐ Behavioural & social sciences ☐ Ecological, evolutionary & environmental sciences

For a reference copy of the document with all sections, see nature.com/documents/nr-reporting-summary-flat.pdf

Life sciences study design

All studies must disclose on these points even when the disclosure is negative.

Sample size	Sample size was determined based on previous experience and was large enough to detect differences between samples with biological significance.
Data exclusions	Animals that died during the experiment were not analysed.
Replication	Results were verified by independent biological repeats as described in the figure legends.
Randomization	All animals in this study were all randomly assigned, and age was matched across groups.
Blinding	No blinding was used as the investigator performed all experiments.

Reporting for specific materials, systems and methods

We require information from authors about some types of materials, experimental systems and methods used in many studies. Here, indicate whether each material, system or method listed is relevant to your study. If you are not sure if a list item applies to your research, read the appropriate section before selecting a response.

Materials & experimental systems

n/a	Involved in the study	
☐	☒	Antibodies
☐	☒	Eukaryotic cell lines
☒	☐	Palaeontology and archaeology
☐	☒	Animals and other organisms
☒	☐	Clinical data
☒	☐	Dual use research of concern
☒	☐	Plants

Methods

n/a	Involved in the study	
☒	☐	ChIP-seq
☒	☐	Flow cytometry
☒	☐	MRI-based neuroimaging

Antibodies

Antibodies used	anti-TSG101 antibody (Abcam ,125011)<br>anti-CD63 antibody(Abcam 213090)<br>anti-ALIX antibody (Abcam , 275377)

	anti-A33 antibody (Cell Signaling Technology, #2449)<br>anti-Calnexin antibody(Thermo, GT1563)
Validation	Antibodies were validated based on the manufacturer's website, which includes relevant literature.

Eukaryotic cell lines

Policy information about cell lines and Sex and Gender in Research

Cell line source(s)	Caco-2 cell line was purchased from Hangzhou Jessimo Biotech.
Authentication	N/A
Mycoplasma contamination	The cell line tested negative for mycoplasma contamination.
Commonly misidentified lines<br>(See ICLAC register)	No commonly misidentified cells lines were used.

Animals and other research organisms

Policy information about studies involving animals; ARRIVE guidelines recommended for reporting animal research, and Sex and Gender in Research

Laboratory animals	Mongolian gerbils were purchased from the Animal Center of Hangzhou Medical College, aged 8 weeks
Wild animals	The study did not involve wild animals.
Reporting on sex	All mice were males.
Field-collected samples	The study did not involve samples collected from the field.
Ethics oversight	This animal study was approved by the Zhejiang Institute for Food and Drug Control, with the experimental animal license number SCXK (Zhe) 2019-0002.

Note that full information on the approval of the study protocol must also be provided in the manuscript.

Plants

Seed stocks	N/A
Novel plant genotypes	N/A
Authentication	N/A
